# Peptide–based artificial cytoskeleton enhances colocalized cascade reactions in cell-like microreactors

**DOI:** 10.1039/d5sc08106h

**Published:** 2026-02-18

**Authors:** Thao P. Doan-Nguyen, Shoupeng Cao, Tsvetomir Ivanov, Katharina Landfester, Lucas Caire da Silva

**Affiliations:** a Department of Physical Chemistry of Polymers, Max Planck Institute for Polymer Research Ackermannweg 10 Mainz 55128 Germany lucas.cairedasilva@mcgill.ca landfester@mpip-mainz.mpg.de; b International Center for Young Scientists, National Institute for Materials Science 1-2-1 Sengen Tsukuba Ibaraki 305-0047 Japan; c College of Polymer Science and Engineering, State Key Laboratory of Polymer Materials Engineering, Sichuan University Chengdu 610065 PR China; d Department of Chemistry, McGill University Montreal Quebec H3A0B8 Canada

## Abstract

We report the formation of an artificial cytoskeleton within droplet-based protocells, which dramatically increases the efficiency of a cascade reaction. The cytoskeleton is formed *via* self-assembly of a short peptide phenylalanine-phenylalanine-methionine (FFM) in the confinement of cell-sized water-in-oil droplets. FFM undergoes coacervation, followed by fiber formation when increasing the pH value from 5.3 to 8, resulting in the generation of a fibrous network within the droplets, resembling a cytoskeleton. This cytoskeleton can bind proteins and enzymes on it, such as bovine albumin serum, glucose oxidase and horseradish peroxidase, resulting in the co-localization of the enzymes on the fiber network, which leads to the enhancement of cascade reaction efficiency. The efficiency of the cascade is even further increased when reducing the size of the microreactors from 86 µm to 49 µm and 31 µm. This artificial cytoskeleton mimics an important feature of the natural cytoskeleton, providing anchorage and colocalization for enzymes involved in cascade reactions.

## Abstract 2

We engineered peptide-based artificial cytoskeletons in droplets to enhance enzyme colocalization and boost cascade reaction efficiency.

## Graphical abstract

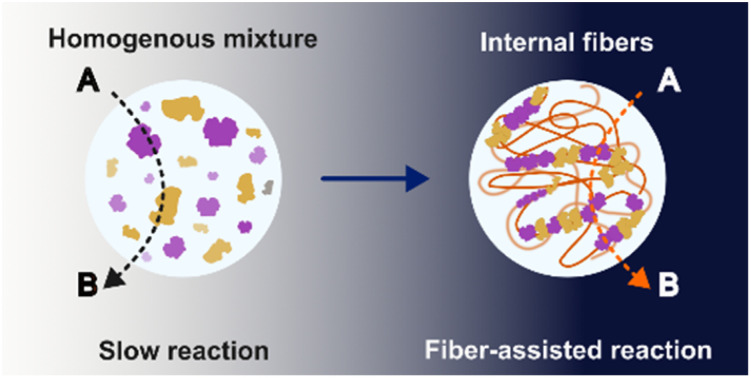

## Introduction

At the dawn of microscopy development, cellular organelles were believed to flow freely in the cell cytoplasm. That was until the exploration of the cytoskeleton, a dynamic network that provides structural support and plays crucial roles in many vital processes of the cell cycle, such as division, transport, signalling, and motility. Efforts in building artificial cells from synthetic building blocks also involve the construction of an artificial cytoskeleton. Artificial cytoskeletons have been assembled from G-actin,^1,2^ tubulin,^1^ DNA,^3,4^ peptides,^4,5^ synthetic polymers,^6^ metal-phenolic network,^7^ among other biomimetic and synthetic building blocks. For instance, metal-phenolic networks as artificial cytoskeleton stabilized liposomes upon exposure to harsh environments, and controlled the permeability and morphology of the vesicles.^7^ A network of nanofibers was assembled within proteinosomes *via* enzymatic dephosphorylation of Nap-FFK(NBD)pY oligopeptide, in order to mimic the cytoskeleton.^5^ The nanofibers were responsible for the morphological transformation of the proteinosomes depending on the concentration of the oligopeptide and the duration during which the fibers grew. Recently, DNA-based cytoskeleton was used as transport tracks to guide the movement of cargo such as lipid vesicles or gold nanoparticles within synthetic cells.^8^ While current studies successfully assembled artificial cytoskeletons, their function is still mostly limited to providing mechanical support, inducing deformation or motility of protocells.

An important role of the natural cytoskeleton is to provide anchorage for organelles and protein molecules in order to facilitate intracellular signaling and reactions.^9^ Many glycolytic enzymes, protein kinases, lipid kinases, phospholipases, and GTPases are known to dock on the eukaryotic cytoskeleton.^10–16^ The interaction of enzymes with the cytoskeleton affects intracellular enzymatic cascades because such binding helps to stabilize metabolons,^17^ which are enzyme complexes that allow the channeling of intermediates from the active site of one enzyme to the others. For instance, the multi-enzyme complex responsible for the *de novo* purine biosynthesis was found to bind to microtubules, while the disruption of microtubules impaired the enzyme complex activity.^18^ In a synthetic system of oil-in-water (o/w) droplets, alkaline phosphatase (ALP), glucose oxidase (GOx) and horseradish peroxidase (HRP) autonomously form clusters at the o/w interface, in response to glucose-6-phosphate (G-6-P), resembling the natural metabolon.^19^ This metabolon formation was attributed to the initial binding of G-6-P and ALP to the Zn^2+^ headgroup of the surfactant used, and followed by a sequential chemotaxis of GOx and HRP. However, developing bio-inspired artificial cell systems that successfully integrate a cytoskeleton to spatially organize molecules, anchor enzymes and enhance cascade efficiency remains a significant challenge. Recently, a polydiacetylene (PDA)-based cytoskeleton was used as a scaffold in coacervate-based artificial cells, which allowed protein reconstitution by bringing two protein subunits in close proximity.^20^ Indeed, luciferase was split into two inactive fragments, LgBiT-c-Raf S233/S259-Histidine and mNeonGreen-SmBiT101-His. Ni^2+^–NTA-modified PDA fibrils allowed co-localization of these protein subunits on the fibrils *via* His Ni–NTA interaction, thus facilitating the reconstitution of luciferase. Nevertheless, the use of the cytoskeleton to enhance cascade efficiency, just like in natural cells, remains largely unexplored in artificial cell systems.

We report here the formation of an artificial cytoskeleton from the self-assembly of a short peptide within droplet-based protocells, which serves as a scaffold to co-localize enzymes catalyzing cascade reactions. Co-localization of enzymes was previously achieved by co-clustering,^21^ or co-encapsulating them in systems such as nanocarriers,^22–25^ virus-like particles,^26–28^ DNA-based cages and scaffolds,^29–31^ micro- and nanogels,^32,33^ liposomes,^34^ polymersomes,^35–37^ dendrimesomes,^38^ coacervates,^39^ hydrogen-bonded organic frameworks,^40^ and metal–organic frameworks.^41^ However, enzyme encapsulation through complex processes and in the presence of incompatible interfaces faces the risk of losing enzyme activity.^42,43^ Sequestering enzymes within coacervate droplets self-assembled under mild conditions offers a versatile strategy for enzyme encapsulation and immobilization. For instance, glucose oxidase and horseradish peroxidase were sequestered inside coacervate subcompartments within polymeric microreactors. The addition of glucose and Amplex red from outside of the microreactors triggered a cascade reaction that resulted in the localized formation of resorufin within the coacervates.^44^ However, coacervates are prone to coalescence and, ultimately, phase separation.^45^ Supramolecular fibers formed from the co-assembly of cyanuric acid-introduced peptide amphiphiles and melamine-modified nitrobenzofurazan were recruited into dextran-rich droplets within a polyethylene glycol-rich continuous phase.^46^ The supramolecules adsorbed GOx and HRP and recruited the enzymes into the droplets, thus enhancing the GOx-HRP reaction rate. Nevertheless, the formation of droplets from liquid–liquid phase separation resulted in broad size distributions, limited size control and a strong tendency to coalesce.

**Fig. 1:**
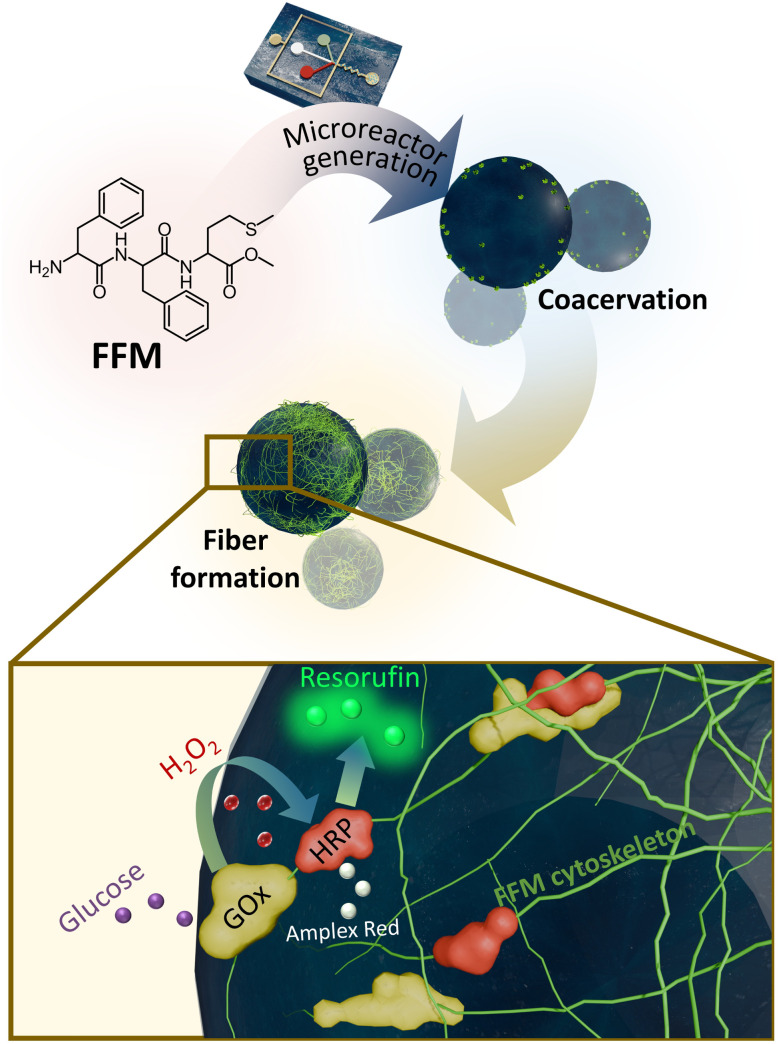
Schematic illustration of the microreactors with artificial cytoskeleton. The cytoskeleton was formed *via* self-assembly of FFM tripeptide in the confinement of cell-sized droplets produced with microfluidics. Coacervation-induced fiber formation occurred within the droplets, resulting in an artificial cytoskeleton that served as a scaffold for co-localization of enzymes in the GOx-HRP cascade.

Alternatively, in this work, phenylalanine-phenylalanine-methionine tripeptide (FFM) underwent self-assembly into fibers in the confinement of water-in-oil droplets at pH 6.5, mimicking the cytoskeleton. The FFM cytoskeleton allowed physical adsorption of enzymes and proteins in mild conditions. When the droplet-based protocells with the cytoskeleton were used as microreactors, the fibers induced co-localization of the two enzymes glucose oxidase (GOx) and horseradish peroxidase (HRP) for the cascade reaction producing resorufin (Fig. 1), leading to a higher rate of resorufin production. Compared to strategies using DNA, polymers, or metal-phenolic networks to build cytoskeletons, the self-assembly of short peptides offers a simplified and straightforward construction process, while ensuring its biological relevance. Compared with longer peptides or proteins, short peptides allow for higher design flexibility and improved synthetic capability. An interesting feature of this artificial cytoskeleton is that the fibers were formed from a coacervation-induced process with coacervates as nucleation sites. This behavior potentially enables a more structured and controllable way of assembling artificial cytoskeletons that closely resemble the natural cytoskeleton and might eventually play structural roles in bio-mimicking processes of artificial cells. The use of water-in-oil droplets stabilized surfactant together with NaCl as an osmotic agent enables the formation of protocells with high stability with no coalescence or Ostwald ripening, suitable for use as microreactors. Moreover, the use of microfluidics for droplet preparation enables monodisperse droplets with precisely controlled sizes, thus allowing control of reaction rates through microreactor size.

## Results and discussion

### Fiber formation from coacervation

**Fig. 2:**
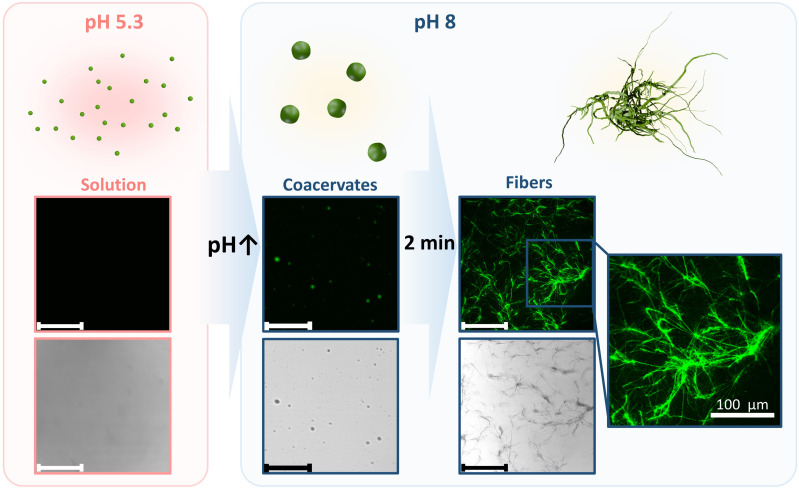
Coacervation-induced fiber formation. Schematic illustration (top) and CLSM micrographs (middle and bottom) of FFM peptide in bulk at pH 5.3 and 8. At pH 5.3, the peptide was soluble at a concentration of 40 mg mL^−1^ in HEPES 5 mM. At pH 8, coacervates were formed *via* liquid–liquid phase separation, followed by rapid fiber formation. Scale bars: 200 µm, unless specified.

Phenylalanine-phenylalanine-methionine (FFM) tripeptide was chosen to build blocks of the artificial cytoskeleton because of its ability to form pH-responsibly fibrous structure, as we reported previously.^47,48^ In order to better microscopically characterize peptide assembly, we synthesized phenylalanine-phenylalanine-nitrobenzoxadiazole (FF-NBD), a fluorescently labeled peptide with a structure close to FFM. When a small amount (3 wt%) of FF-NBD was mixed with FFM, the peptide coacervates and fibers could be observed clearly by confocal laser scanning microscopy (CLSM) at excitation and emission wavelengths of 458 nm and 539 nm, respectively. Below the isoelectric point of FFM (5.7),^48^ the peptide was soluble at a concentration of 40 mg mL^−1^ in HEPES 5 mM, pH 5.3, because its terminal amino group was protonated. Upon increasing pH to 8 by adding HEPES 5 mM, pH 12, the peptide was deprotonated, leading to a decrease in the solvation of FFM. The peptide started to assemble into coacervates first, after that, the coacervates accumulated into fibrous structures of micron size within 2–5 min (Fig. 2 and S11). Our observation shares similarities with the group of Yan, who have reported the existence of a metastable phase during the nucleation of peptides into fibrillar structures.^49^ Solutions of peptides or amino acids went through phase separation into liquid-like solute-rich droplets and a solute-poor continuous phase, before assembling into nanofibrils, which is thermodynamically more favorable. Most short peptides tend to display a strong tendency to form rigid and ordered fiber-like structures, possibly because the highly involved hydrogen bonds guide and facilitate the arrangement and alignment of peptide chains, which favor the formation of solid aggregates at a low-energy state.^49,50^

In previous work, peptide species containing phenylalanine and threonine, such as H-TTFTTFTTF-NH_2_, (TTF)_3_, assembled into nanotubes and exhibited a cross-β architecture determined by *d*-spacings at 4.7 Å and 10.1 Å.^51^ In another study, Lynn and co-authors reported the assembly of the dipeptide H-Asn-Phe-CHO (NF–CHO) and tripeptide H-Asn-Phe-Phe-CHO (NFF–CHO) into fibers. The fiber network exhibited similar morphology to amyloid, and displayed the β-sheet diffractions with characteristic *d*-spacings at 4.7 Å, also consistent with a cross-β structure.^52^ Given the similarity in peptide composition, *i.e.*, tripeptides with diphenylalanine, we propose that FFM fibers are likely to also adopt a cross-β architecture.

### Fabrication of microcompartments with artificial cytoskeleton by pipetting

Our aim was to synthesize microreactors with an artificial peptide-based cytoskeleton. This was achieved by encapsulating FFM peptide into water-in-oil microdroplets while triggering the formation of fibers from peptide self-assembly in confinement. In this arrangement, the liquid interior of the droplets provides a controlled space for fiber formation and structural organization.

**Fig. 3:**
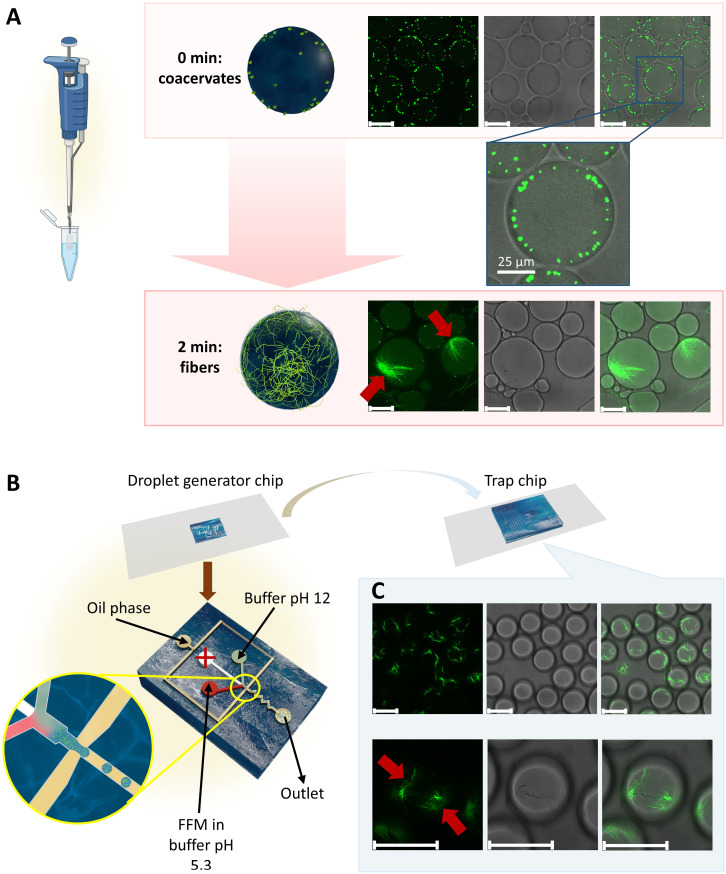
Production of droplet-based protocell with a cytoskeleton. Schematic illustration and CLSM micrographs of droplets containing FFM and FF-NBD peptides formed by (A) pipetting and (B) microfluidics. With microfluidics, the droplets were formed in a droplet generator chip before being transferred into a trap chip for observation. Coacervates were formed and localized close to the interface of water and oil, then became nucleation sites for fiber formation. Red arrows mark the nucleation sites where the fibers started to form and elongate. Scale bars: 50 µm, unless specified.

In order to trigger self-assembly of FFM peptide in the confinement of w/o droplets, a solution of 20 mg mL^−1^ FFM in HEPES buffer (HEPES 5 mM) at pH 5.3 was mixed quickly with an HEPES 5 mM solution at pH 12. Once the pH of the FFM solution was increased to 6.5, it was immediately dispersed in the oil phase by pipetting. Two oil phases were tested for the production of microcompartments: poly(glycerol polyricinoleate) (PGPR) 25 mg mL^−1^ in cyclohexane and fluoroSurfactant (perfluoropolyether/poly(ethylene glycol)-type surfactant or PFPE-PEG-type surfactant) 5 mg mL^−1^ in 3-ethoxy-1,1,1,2,3,4,4,5,5,6,6,6-dodecafluoro-2-(trifluoromethyl)hexane (3 M™ Novec™ 7500 Engineered Fluid, HFE 7500). PGPR in cyclohexane is a common oil phase for preparing water in oil (w/o) emulsions that has been widely used in the literature. HFE 7500 is a fluorinated oil with relatively low interfacial tension with water, thus beneficial for producing w/o droplets. Studies reported the production of homogeneous w/o droplets in microfluidics using the combination of HFE 7500 and fluoroSurfactant.^53–55^ When the aqueous phase containing FFM and FF-NBD (FF-NBD = 3 wt% FFM) was dispersed in cyclohexane, FFM and FF-NBD quickly diffused to the oil phase as detected by fluorescence signals of FF-NBD in the continuous phase (Fig. S12). Because of this diffusion, the concentration of FFM inside the water droplets drastically reduced below the concentration required for nucleation to occur, resulting in no fiber formation. In our solubility test, FFM was not soluble in cyclohexane even at a low concentration of 1 mg mL^−1^. The diffusion of FFM into the oil phase could be attributed to the presence of reverse micelles of PGPR which carried the peptide into the oil. Although the critical micelle concentration (CMC) of PGPR in cyclohexane was not reported, it is estimated to be much lower than the concentration we used (25 mg mL^−1^). Indeed, the CMC of PGPR was ∼1.9 mg mL^−1^ in mixture of heavy and light mineral oils,^56^ and ∼7–13.8 mg mL^−1^ in sunflower oil.^57^ On the contrary, when HFE 7500 was used as the oil phase, the two peptides stayed within the water droplet and formed fibers (Fig. 3a). Interestingly, it could be clearly observed that coacervates were formed at the initial stage and tended to stay close to the water/oil interface (Fig. 3a). These coacervates then became sites of initiation for the growth of fibers, therefore, the fibers tended to have their anchor points close to the water/oil interface. In principle, the observed accumulation of coacervates near the water/oil interface could be explained by both interfacial nucleation and bulk nucleation. In the former case, peptide molecules nucleated at the interface due to a reduced energy barrier for nucleation at such interface. In the latter case, nucleation occurred in the bulk and was followed by sequestration of the coacervates at the water/oil interface due to favorable interfacial adsorption. Fig. 3 and S13 show the nucleation sites, which were the starting points of the fiber elongation. The spatial arrangement of fibers in our system offers control over the formation and organization of the artificial peptide-based cytoskeleton, ensuring a structured and functional network.

### Fabrication of microcompartments with artificial cytoskeleton by microfluidics

The problem with pipetting is that droplets were largely heterogeneous (diameter 45 ± 26 µm), leading to heterogeneity in fiber formation. After screening for good conditions to produce the microcompartments, we optimized the production by using droplet microfluidics. We designed a droplet generator chip that separately injected two aqueous solutions: (i) FFM in buffer at pH 5.3, and (ii) buffer at pH 12 without peptide. These two solutions were mixed quickly at a short junction just before being redispersed in the final oil phase, which led to the formation of water-in-oil droplets with a narrow size distribution (Fig. 3b and S14). Unlike the pipetting method, microfluidics allowed the injection of pH 12 buffer immediately before droplet formation, facilitating fiber nucleation within fully formed, well-defined droplets. The chips were coated with fluoroCoat (Ran Biotechnologies) to prevent the swelling of the PDMS chips upon contact with the fluorinated oil. Droplets generated from the chip were immediately transferred into a trap chip for observation (Fig. S15). Assuming that the volume ratio of the two solutions, buffer at pH 12 and FFM in buffer at pH 5.3, correlated to their flow rates in microfluidics, the pH value inside the droplets is estimated to be 6.5. CLSM micrographs of the w/o emulsion with FFM and fluoroSurfactant 5 mg mL^−1^ in HFE 7500 oil showed homogeneous droplets with fibers formed inside and along their surface (Fig. 3b and Movie S1). The droplets were much more homogeneous (diameter 49 ± 3 µm) compared to the ones prepared by pipetting, and the fiber formation was more prominent. These conditions were then chosen for preparing microreactors.

We observed no difference in droplet stability with or without the peptide fiber. The droplets are highly stable against coalescence and Ostwald ripening due to the presence of 5 wt% fluoroSurfactant in HFE 7500, together with NaCl, which acts as an osmotic agent. No coalescence of droplets or phase separation was observed during time-lapse observations (2–6 hours) (Movie S2).

Interestingly, the fiber formation significantly deformed the droplets. Micrographs of the droplets with and without the fibers (Fig. S16) showed that while droplets without the fibers were perfectly spherical, droplets with the presence of fibers were deformed. Aspect ratio analysis revealed that 60% of fiber-containing droplets were deformed (aspect ratio < 0.95), compared to 0% deformation in droplets without fibers. Previous studies also reported the deformation of protocells caused by an artificial cytoskeleton.^5,58^

**Fig. 4:**
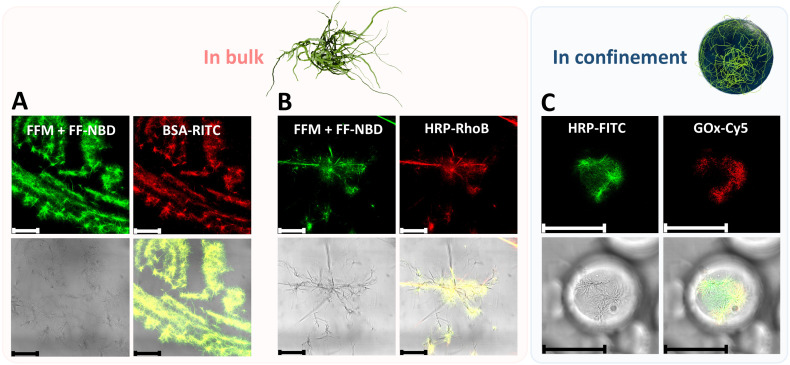
Adsorption of protein and enzymes on FFM + FF-NBD and FFM fibers. CLSM micrographs of (A) BSA-RITC and (B) HRP-RhoB incubated with FFM + FF-NBD fibers in bulk; and (C) HRP-FITC and GOx-Cy5 in droplet microreactors containing FFM fibers. Scale bars: 50 µm.

In addition to the structural effects of the cytoskeleton on the droplets, we found that FFM fibers could be used to anchor proteins and enzymes on them. Indeed, when incubating RITC-labeled BSA (BSA-RITC) and RhoB-labeled horseradish peroxidase (HRP-RhoB) with FFM + FF-NBD fibers in bulk, fluorescent signals of the labeled proteins colocalized well with fluorescent signals of FF-NBD present in the fibers (Fig. 4a and b). That held true when the fibers formed within the microcompartments. In a second experiment, FITC-labeled HRP (HRP-FITC) and Cy5-labeled GOx (GOx-Cy5) were encapsulated with FFM peptide in the microcompartments. It is worth noting that there were no fluorescent signals from the fibers because FF-NBD was not included. Fig. 4c shows the colocalization of HRP-FITC and GOx-Cy5. The fluorescent signals of labeled enzymes co-localized together and co-localized well with the fibers in the bright field. The organization of enzyme molecules on the cytoskeletal network could be mainly attributed to hydrophobic interactions between the hydrophobic side chains of the peptide and the hydrophobic regions of the enzymes. For GOx, in addition to hydrophobic interactions, electrostatic forces are also likely to contribute to the adsorption of enzyme molecules on peptide fibers. Indeed, GOx has an isoelectric point of 4.44,^61^ therefore, is strongly negatively charged inside the microreactors, where pH is estimated to be 6.5. Although pH 6.5 is above FFM's isoelectric point, we expect that the terminal amino group of the peptide would stay facing the aqueous medium and create a positively charged surface of the fibers that could promote GOx adsorption through electrostatic interaction. For HRP, the enzyme has at least 7 isozymes, with isoelectric points ranging from 3.0 to 9.0. We therefore expected that hydrophobic interactions would play a major role in the association of HRP enzyme molecules on the fibers, whereas electrostatic attraction only affects the isozymes whose isoelectric points are below 6.5. In other reports, both the adsorption of GOx on phospholipid monolayer and HRP on cellulosic fiber were dominated by hydrophobic interactions.^59,60^ With this, we have successfully built microreactors with an artificial cytoskeleton that can anchor proteins and enzymes.

### Microreactor for cascade reaction

Our hypothesis is that the artificial cytoskeleton could serve as a scaffold for co-localization of enzymes that catalyze cascade reactions, thus increasing the efficiency of the cascade. To demonstrate this, we chose a cascade reaction of GOx and HRP. In the presence of oxygen, GOx consumed glucose to produce gluconic acid and hydrogen peroxide. Hydrogen peroxide was then consumed by HRP to form resorufin in the presence of Amplex Red. The product resorufin exhibited fluorescent emission at 584 nm.

The reaction was carried out by having the two enzymes and FFM in one aqueous phase at pH 5.3, while the substrates (glucose and Amplex Red) were in another aqueous phase at pH 12. The compositions of the reaction mixtures are shown in Table S3. The two phases were mixed inside the microfluidic chip, therefore, the fiber formation and the cascade reaction were triggered at the same time just before the droplets were formed. In microfluidic systems, mixing is very fast and efficient through diffusion, because it allows for reducing the diffusion distance to as low as several nanometers. While both GOx and HRP are stable in the pH 5 buffer,^62–64^ the mix with the pH 12 stream in microfluidic chips was extremely rapid to produce pH 6.5 within a very short timescale. Despite the short exposure of the enzymes to the pH 12 buffer stream, we do not exclude the possibility that enzyme activity was reduced due to the exposure to the high pH. CLSM micrographs show fluorescent signals of FF-NBD and resorufin, confirming the formation of the artificial cytoskeleton within the microreactors and the production of resorufin from the cascade reaction (Fig. S17).

**Fig. 5:**
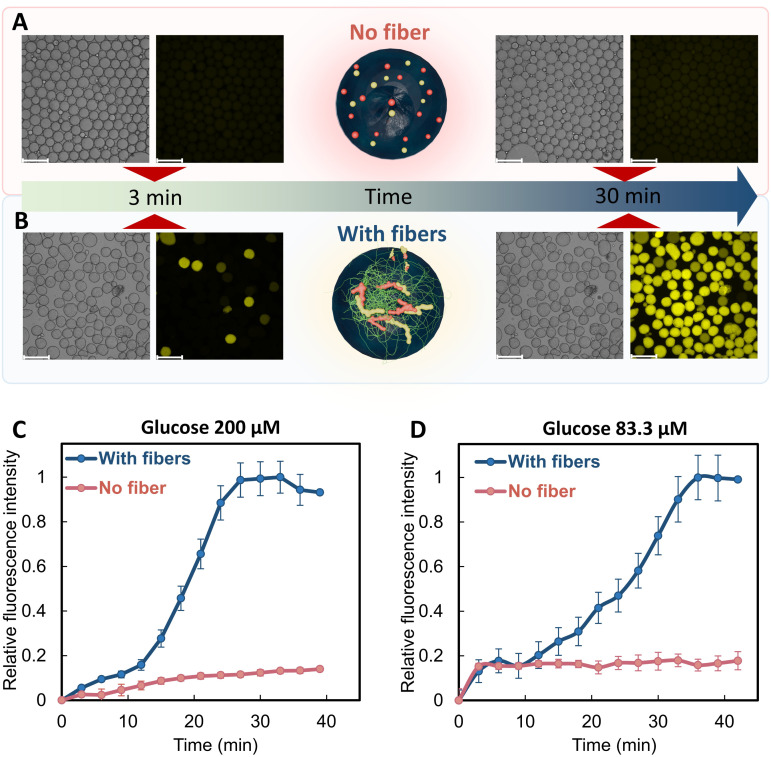
GOx-HRP cascade in microreactors with and without fibers. Micrographs from fluorescence microscope of the GOx-HRP cascade reaction in microreactors (A) without and (B) with the peptide cytoskeleton at 3 and 30 min, at a glucose concentration of 200 µM. (C and D) Kinetics of resorufin production in microreactors with and without fibers at glucose concentrations of 83.3 (D) and 200 µM (C). Scale bars: 200 µm.

When studying the kinetics to compare reaction rates of the microreactors with and without the artificial cytoskeleton, we were surprised that the reaction rate was much faster in the presence of the cytoskeleton. At a glucose concentration of 200 mM, resorufin production plateaued after 27 minutes when fibers were present inside the microreactors. In contrast, in the absence of fibers, the reaction progressed slowly, reaching less than 20% of the relative fluorescence intensity observed with fibers (Fig. 5 and Movie S3). When glucose concentration was reduced to 83.3 mM, the reaction in microreactors with cytoskeleton still reached its plateau after 36 min, whereas resorufin production was still low in the case of no fibers (Fig. S18 and Movie S4). The fibers served as a scaffold for the co-localization of GOx and HRP, which enhanced their proximity to each other and increased the local concentration of the intermediate product hydrogen peroxide, thus improving the efficiency of the cascade. Similar observations were reported for enzymes co-localized in liposomes, nanoparticles and DNA scaffolds.^25,31,34,65,66^

To further demonstrate the effect of the fibers on the reaction rate, a control experiment was performed with the GOx-HRP cascade reaction occurring in buffer solutions with the presence or absence of the peptide fibers. The reaction was performed at pH 6.5, similar to the estimated pH in the droplet microreactors. Two calibration curves of resorufin concentration against fluorescence intensity were obtained at pH 6.5 in the presence or absence of FFM fibers. With the presence of FFM fibers, resorufin production plateaued after only 30 seconds. After reaching plateau, a decrease in fluorescence signal of resorufin was observed. This behavior is attributed to the oxidation of resorufin over time. In addition, the gradual precipitation of FFM fibers in the buffer solution may contribute to the decrease of fluorescence signal, possibly due to sequestration of resorufin on the fibers or increased light scattering effects. In contrast, in the absence of the fibers, the reaction reached its plateau after 5.5 min (Fig. S19). These results are consistent with the effect of fibers on GOx-HRP reaction in the droplet microreactors, where the fibers produced co-localization of the enzymes, increased proximity, and reduced intermediate losses, thus enhancing the reaction efficiency. The higher rates of the reaction in buffer solutions compared to the reaction in droplets can be attributed to the improved oxygen availability in the case of the buffer solution because GOx is highly dependent on dissolved oxygen to produce hydrogen peroxide. Furthermore, it could also partially result from the reduced enzyme activity in the case of the reaction in droplets, where the enzymes were shortly exposed to a buffer pH 12 stream.

### Microreactor size affects reaction rates

The effect of the artificial cytoskeleton on reaction rate was proven to be very prominent. We later on found that reaction rates could be even further boosted by decreasing the size of the microreactors with the artificial cytoskeleton. In order to do that, microreactors with different sizes were prepared by modifying the flow rates of the outer fluid (*i.e.* the continuous phase) in microfluidics. Smaller droplets were formed with higher flow rates of the outer fluid.^44^ This is because when increasing the velocity of the continuous phase, larger shear stresses are exerted on the water/oil interface, breaking the droplets into smaller sizes.^67,68^ In our case, droplets with an average diameter of 86 ± 4 µm were formed when the outer fluid was injected at 400 µL h^−1^, whereas 49 ± 3 µm droplets were obtained when the outer fluid flow rate was 1000 µL h^−1^ (Table S4). FFM fibers were formed regardless of the size of the microreactors (Fig. S13).

**Fig. 6:**
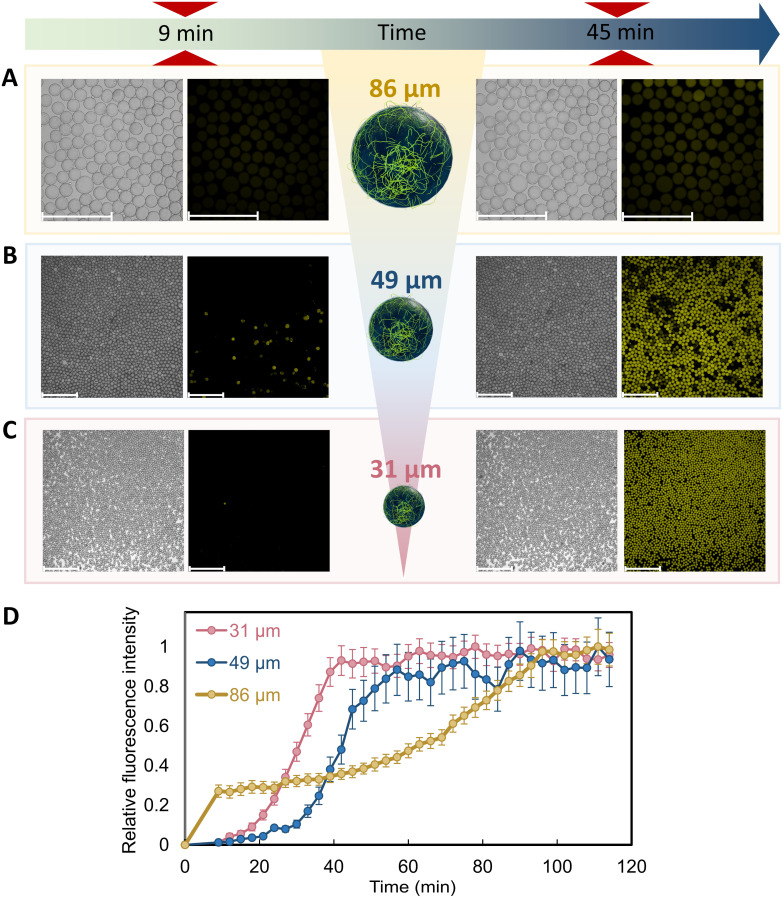
GOx-HRP cascade in microreactors of different sizes. Micrographs from fluorescence microscope of the GOx-HRP cascade reaction in microreactors with diameters of (A) 86 ± 4 µm, (B) 49 ± 3 µm and (C) 31 ± 2 µm with the peptide cytoskeleton, at 9 and 45 min. (D) Kinetics of resorufin production in microreactors of different diameters with cytoskeleton. The glucose concentration was 5 mM in all cases. Scale bars: 500 µm.

Cascade reaction with GOx and HRP was carried out in order to compare the reaction rates between microreactors with the artificial cytoskeleton at different sizes. At 10 minutes of the reaction, a higher production of resorufin was observed for microreactors with a diameter of 86 µm, compared to the ones with diameters of 31 and 49 µm. This could be attributed to the higher initial dissolved oxygen present in larger droplets compared to smaller ones, since GOx is highly dependent on dissolved oxygen to produce hydrogen peroxide. After the initial stage, although concentrations of all substrates and enzymes were kept equal between microreactors of different sizes, resorufin production was faster for smaller microreactors with a cytoskeleton. Indeed, at a very low concentration of glucose (5 mM), resorufin production reached its plateau after 42, 57 and 96 min for microreactors with a diameter of 31 ± 2, 49 ± 3 and 86 ± 4 µm, respectively (Fig. 6 and Movie S5). This can be attributed to the difference in fiber formation between the large and small droplets. Fibers tended to spread throughout most of the volume, or the whole volume of the small droplets (49 µm). In contrast, in the case of the large droplet (86 µm), the fibers occupied only a portion of the volume (Fig. S13). This difference likely arises from the relative water/oil interfacial area. Small droplets have a higher surface-area-to-volume ratio (see calculations in Table S5), which favors coacervate localization at the interface and promotes fiber nucleation. Similar to a natural cytoskeleton, our artificial filamentous network subdivides the droplet's interior, creating micro-compartments within the microreactors. The more the fibers spread, the better the microreactors were micro-compartmentalized, reducing the dead volume where substrates could not meet the enzymes attached to the fibers. Furthermore, because the enzymes co-localized on the fibers, the fibers became reaction sites. In large droplets, because the fibers only partially covered the droplet volume, reactant molecules (*i.e.*, glucose, Amplex Red) needed to diffuse through a considerable distance to reach their catalytic sites. For example, a molecule positioned near the droplet interface would face a long diffusion distance if the fibers were localized on the opposite side. In small droplets, the fibers spread throughout most of the volume, resulting in shorter distances between the reactant molecules and the enzyme-loaded fibers. The shorter diffusion time yielded an enhanced reaction rate. This relation between the diffusion time and reaction rate was also reported in a previous study, in which Wei *et al.* compared the reaction rates between droplet microreactors of different sizes.^69^

## Conclusion

An artificial cytoskeleton was constructed within the confinement of water-in-oil droplets that were used as microreactors. The artificial cytoskeleton was formed from the self-assembly of phenylalanine-phenylalanine-methionine tripeptide when increasing the pH value from 5.3 to 8. The artificial cytoskeleton allows adsorption of protein (bovine albumin serum) and enzymes (glucose oxidase and horseradish peroxidase) on it, thus serving as a scaffold for co-localization of enzymes for cascade reactions. Using the microreactors with the cytoskeleton, the efficiency of the cascade GOx-HRP was dramatically enhanced compared to the reaction in microreactors without the cytoskeleton, due to the increased proximity of the two catalysts. When the diameter of the microreactors with cytoskeleton was decreased from 86 to 49 and 31 µm, the efficiency of the GOx-HRP cascade was further improved due to the higher surface-area-to-volume ratios for small droplets. The increase in total interfacial area resulted in an increase in nucleation sites, which led to more coverage of the fibers over the volume of the microreactors. Our system mimics an important characteristic of the eukaryotic cytoskeleton, which is anchoring enzymes for boosting enzymatic cascades. These findings highlight the potential of peptide fibers to regulate catalytic activity in cell-like systems by mimicking biological strategies for organizing enzymatic processes. The approach offers a robust and versatile framework for designing functional microreactors with biomimetic architectures.

## Author contributions

T. P. D.-N., S. C. and T. I.: investigation, methodology, validation, analysis, writing, and reviewing. K.L., L. C. S.: conceptualization, methodology, funding acquisition, supervision, writingconceptualization, methodology, funding acquisition, supervision, writing, and reviewing.

## Conflicts of interest

There are no conflicts to declare.

## Supplementary Material

SC-017-D5SC08106H-s001

SC-017-D5SC08106H-s002

SC-017-D5SC08106H-s003

SC-017-D5SC08106H-s004

SC-017-D5SC08106H-s005

SC-017-D5SC08106H-s006

## Data Availability

The data supporting this study are available within the article and in the accompanying supplementary information (SI). Supplementary information is available. See DOI: https://doi.org/10.1039/d5sc08106h.
