# Supplementary material for: Peptide–based artificial cytoskeleton enhances colocalized cascade reactions in cell-like microreactors
Source: Chem Sci. 2026 Feb 18;17(15):7707–16. doi: 10.1039/d5sc08106h (PMC12934255; doi:10.1039/d5sc08106h)
Supplement: SC-017-D5SC08106H-s006 [file SC-017-D5SC08106H-s006.pdf]

Supplementary Information (SI) for Chemical Science.
This journal is © The Royal Society of Chemistry 2026

Supporting Information

Peptide–Based Artificial Cytoskeleton Enhances Colocalized Cascade Reactions in Cell-Like Microreactors

Thao P. Doan-Nguyen,[a,b] Shoupeng Cao,[a,c] Tsvetomir Ivanov,[a] Katharina Landfester,[a] and Lucas Caire da Silva*[a,d]*

[a] Dr. T. P. Doan-Nguyen, Dr. S. Cao, T. Ivanov, Dr. L. Caire da Silva, Prof. Dr. K. Landfester
Department of Physical Chemistry of Polymers
Max Planck Institute for Polymer Research
Ackermannweg 10, 55128 Mainz, Germany
E-mail: lucas.cairedasilva@mcgill.ca, landfester@mpip-mainz.mpg.de
[b] Dr. T. P. Doan-Nguyen
International Center for Young Scientists
National Institute for Materials Science
1-2-1 Sengen, Tsukuba, Ibaraki 305-0047, Japan
[c] Dr. S. Cao
College of Polymer Science and Engineering, State Key Laboratory of Polymer Materials Engineering
Sichuan University
Chengdu 610065, PR China
[d] Dr. L. Caire da Silva
Department of Chemistry
McGill University
801 Sherbrooke St. West, Montreal, Canada

1. EXPERIMENTAL SECTION

1.1. Materials

3-ethoxy-1,1,1,2,3,4,4,5,5,6,6,6-dodecafluoro-2-(trifluoromethyl)hexane (3M™ Novec™ 7500 Engineered Fluid, HFE 7500), FluoroSurfactant (008-FluoroSurfactant) and FluoroCoat were purchased from RAN Biotechnologies. Amplex Red, Glucose Oxidase from *Aspergillus niger* (GOx, Type X-S, lyophilized powder, 100,000 – 250,000 units/g solid, G7141-50KU), peroxidase from horseradish (HRP, Type VI, essentially salt-free, lyophilized powder, ≥250 units/mg solid, P8375-5KU), sodium hydroxide (NaOH, 97%), HEPES buffer (1M solution), sodium chloride (NaCl, 99%), D-(+)-glucose (99.5%), cyclohexane (99%) were purchased from Sigma Aldrich. Polyglycerol polyricinoleate (PGPR, Danisco, Germany), resorufin (95%, TCI), polydimethylsiloxane (PDMS, SYLGARD™ 184 Silicone Elastomer Kit, Dow Corning) and curing agent (SYLGARD™ 184 Silicone Elastomer Kit, Dow Corning) were used as received. Ultrapure water was used throughout all experiments.

1.2. Synthesis of phenylalanine-phenylalanine-methionine (FFM) and phenylalanine-phenylalanine—nitrobenzoxadiazole (FF-NBD) peptides

FFM and FF-NBD were synthesized following our previous work.[1]

1.2.1. FFM

This compound was synthesized in a four-step reaction (**Figure S1**).

(**i**) Synthesis of BOC-F-M-OMe: N-(tert-butoxycarbonyl)-*L*-phenylalanine (Boc-F-OH) (1.45 g, 5.5 mmol), HBTU (2.08 g, 5.5 mmol) and HOBt (0.75 g, 5.5 mmol) were dissolved in DMF (10 mL) in a round-bottom flask and the mixture was stirred with a magnetic stirrer. N,N-diisopropylethylamine (DIPEA) (1.75 mL, 10 mmol), and L-methionine methyl ester hydrochloride (1 g, 5 mmol) were added with an interval of 1 min, respectively, and the reaction mixture was stirred for 24 h at room temperature. The reaction mixture was poured into 250 mL of water. White precipitate was collected by filtration and washed with water. The crude product was dried in an oven at 40 °C overnight, which produced 1.26 g of white solid and was characterized by NMR spectroscopy (**Figure S2**). ^{1}H NMR (300 MHz, $CDCl_3$) δ 7.42 – 7.14 (m, 5H), 6.56 (d, J = 7.7 Hz, 1H), 4.99 (s, 1H), 4.66 (m, 1H), 4.37 (d, J = 7.3 Hz, 1H), 3.73 (s, 3H), 3.16 – 3.00 (m, 2H), 2.42 (t, 2H), 2.07 (s, 3H), 1.44 (s, 9H). ^{13}C NMR (75 MHz, $CDCl_3$) δ 171.74, 171.04, 155.35, 136.41, 129.34, 128.71, 127.02, 80.37, 55.79, 52.50, 51.56, 38.06, 31.59, 29.69, 28.25, 15.38.

(**ii**) Synthesis of NH_2-F-M-OMe: The intermediate compound (1.2 g) was dissolved in 9 mL of 4 M hydrogen chloride solution in dioxane for protection. After stirring for 3 h, the solvent

was evaporated on a rotary evaporator, yielding an oily residue. Diethyl ether was added to the flask, and the content was gently stirred. A white precipitate formed and was separated by centrifugation, which produced around 1.13 g of white product. The product was further characterized by NMR spectroscopy techniques (**Figure S3**). ^{1}H NMR (300 MHz, DMSO-d_6) δ 9.10 (d, *J* = 7.6 Hz, 1H), 8.36 – 8.27 (m, 2H), 7.40 – 7.17 (m, 5H), 4.52 – 4.37 (m, 1H), 4.10 (s, 1H), 3.76 – 3.64 (m, 1H), 3.63 (s, 3H), 3.49 (s, 1H), 3.14 (m, 1H), 2.99 (m, 1H), 2.05 (s, 3H), 2.01 – 1.78 (m, 2H). ^{13}C NMR (75 MHz, DMSO) δ 171.46, 168.19, 134.81, 129.53, 128.43, 127.07, 53.18, 52.10, 50.80, 36.67, 30.41, 29.26, 14.40.

(**iii**) Synthesis of BOC-F-F-M-OMe: Then, N-(tert-butoxycarbonyl)-*L*-phenylalanine (Boc-F-OH) (0.9 g, 3.3 mmol), HBTU (1.26 g, 3.3 mmol) and HOBt (0.45 g, 3.3 mmol) were dissolved in DMF (7 mL) in a round-bottom flask and the mixture was stirred with a magnetic stirrer. N,N-diisopropylethylamine (DIPEA) (1.05 mL, 6 mmol), and the above intermediate (1.04 g, 3 mmol) were added with an interval of 1 min, respectively, and the reaction mixture was stirred for 24 h at room temperature. The reaction mixture was poured into 100 mL of water. White precipitate was collected by filtration and washed with water. The crude product was dried in an oven at 40 °C overnight, which produced 1.5 g of white solid and was characterized by NMR spectroscopy (**Figure S4**). ^{1}H NMR (300 MHz, Chloroform-*d*) δ 7.38 – 7.05 (m, 10H), 6.72 – 6.32 (m, 2H), 4.84 (d, *J* = 6.6 Hz, 1H), 4.74 – 4.54 (m, 2H), 4.31 (d, *J* = 6.0 Hz, 1H), 3.73 (s, 3H), 3.19 – 2.89 (m, 4H), 2.40 (t, *J* = 7.4 Hz, 2H), 2.07 (s, 4H), 1.91 (m, 1H), 1.36 (s, 9H).

(**iv**) Synthesis of FFM: Then, the above intermediate compound (1.45 g) was dissolved in 10 mL of 4 M hydrogen chloride solution in dioxane for protection. After stirring for 3 h, the solvent was evaporated on a rotary evaporator, yielding an oily residue. Diethyl ether was added to the flask, and the content was gently stirred. A white precipitate formed and was separated by centrifugation, which produced around 1.2 g of white solid. The product was further characterized by NMR spectroscopy and GC-MS (**Figure S5**). ^{1}H NMR (400 MHz, DMSO-d_6) δ 8.93 (d, *J* = 8.1 Hz, 1H), 8.67 (d, *J* = 7.7 Hz, 1H), 8.11 (s, 2H), 7.41 – 7.17 (m, 10H), 4.62 (m, 1H), 4.45 (m, 1H), 4.01 (s, 1H), 3.63 (s, 3H), 3.15 (m, 1H), 3.06 (m, 1H), 2.90 (m, 2H), 2.05 (s, 3H), 2.03 – 1.85 (m, 2H). ^{13}C NMR (75 MHz, DMSO) δ 171.92, 170.83, 167.82, 137.40, 134.77, 129.69, 129.25, 128.48, 128.35, 128.11, 126.98, 126.37, 66.31, 54.27, 53.14, 51.96, 50.92, 37.44, 36.63, 30.43, 29.47, 14.50. MS(EI): calculated for $C_{24}H_{31}N_3O_4S$: 457.20 (M^+); MALDI-TOF found 458.2245 ($M+H^+$).

1.2.2. NH_2-FF-NBD (FF-NBD)

NH_2-NBD was synthesized according to the literature.[2] The synthesis of NH_2-FF-NBD was carried out with adaptive procedures compared to the literature.[1, 3] The synthetic procedure is illustrated in **Figure S6**.

Boc-F-NBD: N-(tert-butoxycarbonyl)-L-phenylalanine (Boc-F-OH) (450 mg, 1.65 mmol), HBTU (624 mg, 1.65 mmol) and HOBt (225 mg, 1.65 mmol) were dissolved in DMF (5 mL) in a round bottom flask, and the mixture was stirred with a magnetic stirrer. N,N-diisopropylethylamine (DIPEA) (525 µL, 3 mmol), and NH_2-NBD (400 mg, 1.5 mmol) were added at 1 min intervals, respectively, and the reaction mixture was stirred for 24 h at room temperature. The reaction mixture was poured into 100 mL of water. The precipitate was collected by filtration and washed with water. The crude product was dried and further purified via column chromatography to give 703 mg brown solid, which was characterized by NMR spectroscopy (**Figure S7**). ^{1}H NMR (400 MHz, DMSO-d_6) δ 9.36 (s, 1H), 8.54 (d, 1H), 8.16 (t, 1H), 7.30 – 7.09 (m, 5H), 6.95 (d, 1H), 6.45 (d, 1H), 4.08 (m, 1H), 3.60 – 3.35 (m, 4H), 3.01 – 2.87 (m, 1H), 1.27 (s, 9H). ^{13}C NMR (101 MHz, DMSO-d_6) δ 172.69, 155.68, 145.86, 144.91, 138.58, 129.57, 128.45, 126.60, 99.75, 78.48, 56.27, 38.72, 37.86, 30.07, 28.54.

NH2-F-NBD: The intermediate compound was dissolved in 6 mL of 4 M hydrogen chloride solution in dioxane for protection. After stirring for 3 h, the solvent was evaporated on a rotary evaporator to give an oily residue. Diethyl ether was added to the flask and the contents were stirred gently. A white precipitate formed and was separated by centrifugation, yielding about 601 mg of a yellow product. The product was characterized by NMR spectroscopy (**Figure S8**). ^{1}H NMR (400 MHz, DMSO-d_6) δ 9.34 (s, 1H), 8.81 (s, 1H), 8.56 (d, 1H), 8.35 (s, 3H), 7.33 – 7.18 (m, 6H), 6.44 (d, 1H), 3.96 (d, 1H), 3.77 – 3.63 (m, 1H), 3.53 – 3.45 (m, 3H), 3.23 – 2.81 (m, 3H). ^{13}C NMR (101 MHz, DMSO-d_6) δ 168.88, 145.63, 138.42, 135.42, 129.87, 128.95, 127.55, 99.71, 72.63, 70.99, 60.65, 54.08, 38.72, 37.26.

Boc-FF-NBD: N-(tert-butoxycarbonyl)-L-phenylalanine (Boc-F-OH) (450 mg, 1.65 mmol), HBTU (624 mg, 1.65 mmol) and HOBt (225 mg, 1.65 mmol) were dissolved in DMF (5 mL) in a round-bottom flask and the mixture was stirred with a magnetic stirrer. N,N-diisopropylethylamine (DIPEA) (525 µL, 3 mmol), and NH_2-F-NBD (590 mg, 1.44 mmol) were added at 1 min intervals, respectively, and the reaction mixture was stirred for 24 h at room temperature. The reaction mixture was poured into 100 mL of water. The precipitate was collected by filtration and washed with water. The crude product was dried and further purified via column chromatography to give 403 mg brown solid, which was characterized by NMR spectroscopy (**Figure S9**). ^{1}H NMR (400 MHz, DMSO-d_6) δ 9.34 (s, 1H), 8.56 (d, 1H), 8.24 (d, 1H), 8.11 – 7.97 (m, 1H), 7.30 – 7.06 (m, 11H), 6.91 (d, 1H), 6.43 (d, 1H), 4.48 (d, 1H),

4.26 – 4.05 (m, 1H), 3.47 (s, 2H), 3.11 – 2.78 (m, 4H), 2.72 – 2.55 (m, 2H), 1.32 (d, 9H). ^{13}C NMR (101 MHz, DMSO-d_6) δ 174.07, 171.87, 171.79, 155.60, 138.51, 138.47, 137.88, 129.67, 129.55, 128.68, 128.60, 128.50, 128.43, 126.76, 126.59, 78.64, 78.51, 70.92, 68.51, 64.18, 56.30, 55.60, 54.28, 28.63, 28.56.

NH2-FF-NBD: The intermediate compound (100 mg) was dissolved in 6 mL of 4 M hydrogen chloride solution in dioxane for protection. After stirring for 3 h, the solvent was evaporated on a rotary evaporator to give an oily residue. Diethyl ether was added to the flask and the contents were stirred gently. A white precipitate formed and was separated by centrifugation, yielding about 85 mg of a yellow product. The product was characterized by NMR spectroscopy. ^1H NMR (400 MHz, DMSO-d_6) δ 9.33 (s, 1H), 8.96 (d, J = 8.0 Hz, 1H), 8.54 (d, J = 8.9 Hz, 1H), 8.43 (s, 1H), 8.18 – 8.12 (m, 3H), 7.30 – 7.20 (m, 11H), 6.44 (d, J = 9.0 Hz, 1H), 4.47 (td, J = 8.1, 6.0 Hz, 1H), 4.03 (s, 1H), 3.47 (d, J = 2.3 Hz, 2H), 3.16 – 2.80 (m, 6H). ^{13}C NMR (101 MHz, DMSO-d_6) δ 171.31, 168.31, 137.78, 135.19, 130.02, 129.96, 129.61, 129.05, 128.90, 128.63, 127.69, 127.54, 126.89, 66.83, 55.00, 53.67, 53.61, 37.30. MALDI MS(EI): calculated for $C_{26}H_{27}N_7O_5$: 517.2074 (M+); found 518.2239 ($M+H^+$) (**Figure S10**)).

1.3. Preparation of microcompartments with an artificial cytoskeleton

1.3.1. Pipetting

An oil phase was prepared by dissolving 50 mg of FluoroSurfactant in 1 mL of HFE 7500 oil. For the aqueous phase, 20 µL of 20 mg/mL FFM solution was mixed with 0.6 µL of 20 mg/mL FF-NBD solution and 10 µL of 14.4 mg/mL NaCl solution in HEPES 5mM at pH 5.3. Then 50 µL of HEPES 5mM aqueous solution at pH 12 was quickly mixed into the aqueous phase, which was immediately dispersed into the oil phase by pipetting.

1.3.2. Microfluidics

The w/o emulsions were also produced using a droplet-based microfluidic technique. Microfluidic chips for droplet production and observation were prepared using photolithography as described below.

Chip design

The droplet generator chip and trap chip were designed using LibreCAD software (**Figure S11, Figure S12**). The chip designs were then printed on film masks with darkfield polarity to be used for photolithography.

Photolithography

A silicon wafer was washed and incubated at 150 °C for 10 min to eliminate impurities and moisture. The wafer was left to cool down to room temperature before being coated with SU-8 3050 photoresist (MicroChem) with a spin coater at 3500 rpm for 30 s to obtain a coating

thickness of 45 μm. The wafer coated with photoresist was then processed with soft-baking, including pre-baking on a hot plate at 65 °C for 1 min, baking at 95 °C for 14 min, and then cooling at 65 °C for 1 min before cooling at room temperature. After that, the photoresist-coated wafer was aligned with the film mask containing chip designs in a mask aligner (UV-KUB3) before being exposed to UV light with an intensity of 35 mW/cm^2 for 6.4 s. The UV light triggered a cross-link reaction and hardening of the photoresist following the chip designs in the film mask. Post-exposure baking was then carried out on the wafer, including pre-baking on a hot plate at 65 °C for 1 min, baking at 95 °C for 4 min, and then cooling at 65 °C for 1 min before cooling at room temperature. The remaining photoresist, which was unexposed to UV light, was removed by washing with SU-8 developer solution for 7 min, followed by washing with isopropanol and drying with pressurized air. The wafer was processed with a final hard-baking in an oven at 150 °C for 30 min.

Chip production

Polydimethylsiloxane (PDMS) microfluidic chips were produced from the silicon wafer with channel molds obtained from the photolithography process. Liquid PDMS was mixed with a curing agent at 9:1 weight ratio. The polymer mixture was degassed in a desiccator with a vacuum pump until all air bubbles were eliminated. 40 mg of the mixture was then poured into a glass petri dish (100 mm in diameter) containing the silicon wafer with channel molds at the bottom before being heated in an oven at 80 °C for 2 h.

PDMS replicas of the channel molds were cut using a scalpel and separated from the silicon wafer. The inlets and outlets of the PDMS replicas were pierced with a biopsy punch. A glass slide (25 x 50 mm) was cleaned with ethanol and dried with pressurized air. The surface of the PDMS replica with channels on it and the glass side were activated in a plasma cleaner at 20% power for 30 s. The PDMS replica was then immediately bonded to the activated glass slide by gently pressing them together.

Chip coating

Coating of microfluidic channels was carried out in order to protect PDMS channels from HFE 7500 fluorinated oil, which is incompatible with PDMS and causes deformation to the channels. FluoroCoat solution was filtered using a syringe filter (PTFE, 5 μm pore size). The filtered coating solution was then injected into the channels of the microfluidic chips, reacted with the channels for 30 s and then was flushed out using a vacuum pump. The remaining unreacted coating solution was removed from the channels by rinsing with HFE 7500 oil using the vacuum pump.

Production of microcompartments

To produce w/o droplets, 2 aqueous phases and 1 oil phase were prepared. The compositions of these phases and the flow rate for each phase in the microfluidic setup are shown in **Table S1** and **Table S2**. Inner fluid 1 and inner fluid 2 were prepared and injected separately but were mixed inside the droplet generator chip to become the aqueous phase of the emulsion.

Inner fluid 1, inner fluid 2, and outer fluid were drawn into 1 mL syringes which connected to inlets of the droplet generator chip through microtubing (PTFE, Adtech Polymer Engineering). The flow rates of the inner and outer fluids inside the chip were controlled using a syringe pump. The chip was placed under a microscope lens equipped with a high-speed camera for observation. The resulting droplets produced from the droplet generator chip were directly collected into a pipette tip connected to the chip outlet, and then injected into the inlet of the trap chip for further observation on microscopes.

1.4. Enzymatic cascade reactions in microreactors

The cascade reaction producing resorufin involves two enzymes, glucose oxidase and horseradish peroxidase. The reaction was performed on-chip, together with the production of the microreactors. The enzymes were injected in one channel (inner fluid 1) whereas the substrates for the reaction were injected in another channel (inner fluid 2). Inner fluids 1 and 2 were mixed inside the droplet generator chip very shortly before the droplet microreactors were formed. The compositions of the inner and outer fluids are shown in **Table S3**.

Microreactors of different sizes were produced by modifying the flow rates of the outer fluids. Flow rates to produce microreactors with diameters 31, 49 and 86 μm are shown in **Table S4**.

1.5. Enzymatic cascade reactions in buffer solutions

The cascade reaction producing resorufin, which involves two enzymes, glucose oxidase and horseradish peroxidase, was also performed in buffer solutions. First, FFM fibers were formed by mixing a solution of FFM in HEPES 5 mM, pH 5, with a solution of HEPES 5 mM, pH 12, at a final FFM concentration of 4.15 mg/mL and a pH of 6.5. HRP (3 μg/mL) and GOx (15 μg/mL) were added to the dispersion containing FFM fibers. Subsequently, glucose (200 mM) and Amplex Red (0.058 mM) were added to the mixture, whose fluorescence intensity was measured every 30 seconds with a plate reader (EnSight, PerkinElmer Japan) (excitation: 530 nm, emission: 590 nm). For the control sample without FFM fibers, a solution of HEPES 5 mM, pH 6.5, was used instead of the mixture of FFM fibers at pH 6.5.

Resorufin concentrations were calculated from fluorescence intensity using the relevant calibration curves obtained in section 1.6 below.

1.6. Calibration curve of resorufin in buffer solutions

Two calibration curves were made by measuring fluorescence intensity of resorufin solutions at different concentrations in HEPES 5 mM at pH 6.5 in the absence or presence of FFM fibers. In the latter case, the final concentration of FFM was 4.15 mg/mL. Fluorescence intensity was measured with a plate reader (EnSight, PerkinElmer Japan) (excitation: 530 nm, emission: 590 nm).

1.7. Analytical tools

The production of microreactors with microfluidics was observed with an inverted microscope (Leica DMi 8) equipped with a high-speed camera (Vision Research Phantom VEO 710). Micrographs were acquired with an HC PL FLUOTAR 10x/0.30 or an N PLAN 5x/0.12 dry objective. Cascade reaction GOx-HRP was monitored by acquiring frames every 3 min for 0.5 – 2 h.

Micrographs of coacervates, fibers and microreactors were acquired with a confocal laser scanning microscopy (CLSM, Leica TCS SP5 II) with a HC PL APO CS2 63.0x1.20 UV or HCX IRAPO L 25.0x0.95 water immersion objective. Fluorescence signals were obtained with the following wavelengths: resorufin (excitation: 561 nm, emission: 570 – 640 nm), FF-NBD (excitation: 458 nm, emission: 484 – 550 nm), bovine serum albumin-RITC (BSA-RITC) (excitation: 561 nm, emission: 600 – 660 nm), HRP-RhoB (excitation: 561 nm, emission: 565 – 609 nm), HRP-FITC (excitation: 488 nm, emission: 496 – 577 nm), GOx-Cy5 (excitation: 633 nm, emission: 637 – 743 nm). The microscopes were controlled using Las X software and the high-speed camera was controlled by Phantom Camera Control Application PCC 2.6, Vision Research. Acquired micrographs were analyzed with ImageJ, droplet sizes and aspect ratios were measured from micrographs using SemAfore 5.21 software. Fluorescence intensity of resorufin in buffer solutions was measured with a plate reader (EnSight, Perkin Elmer Japan) (excitation 530 nm, emission: 590 nm).

2. SUPPORTING DATA

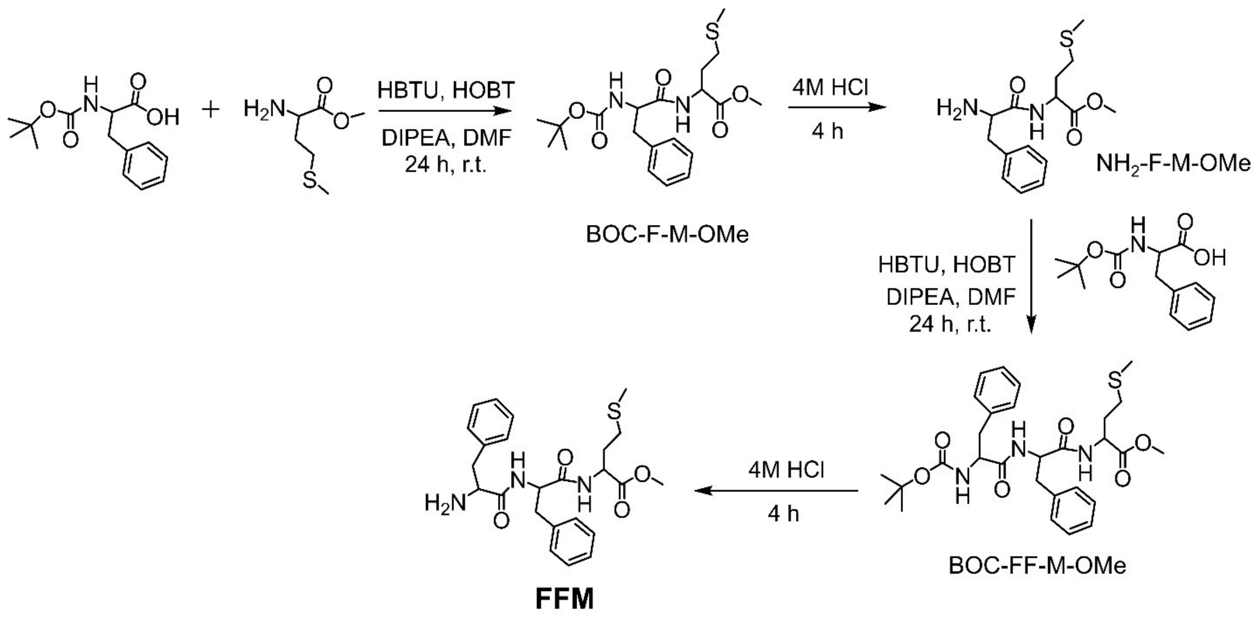

Figure S1. Synthesis of FFM. Synthesis route towards diphenylalanine-based derivatives via simply multi-step reaction according to literature report with slight modifications.[1, 3]

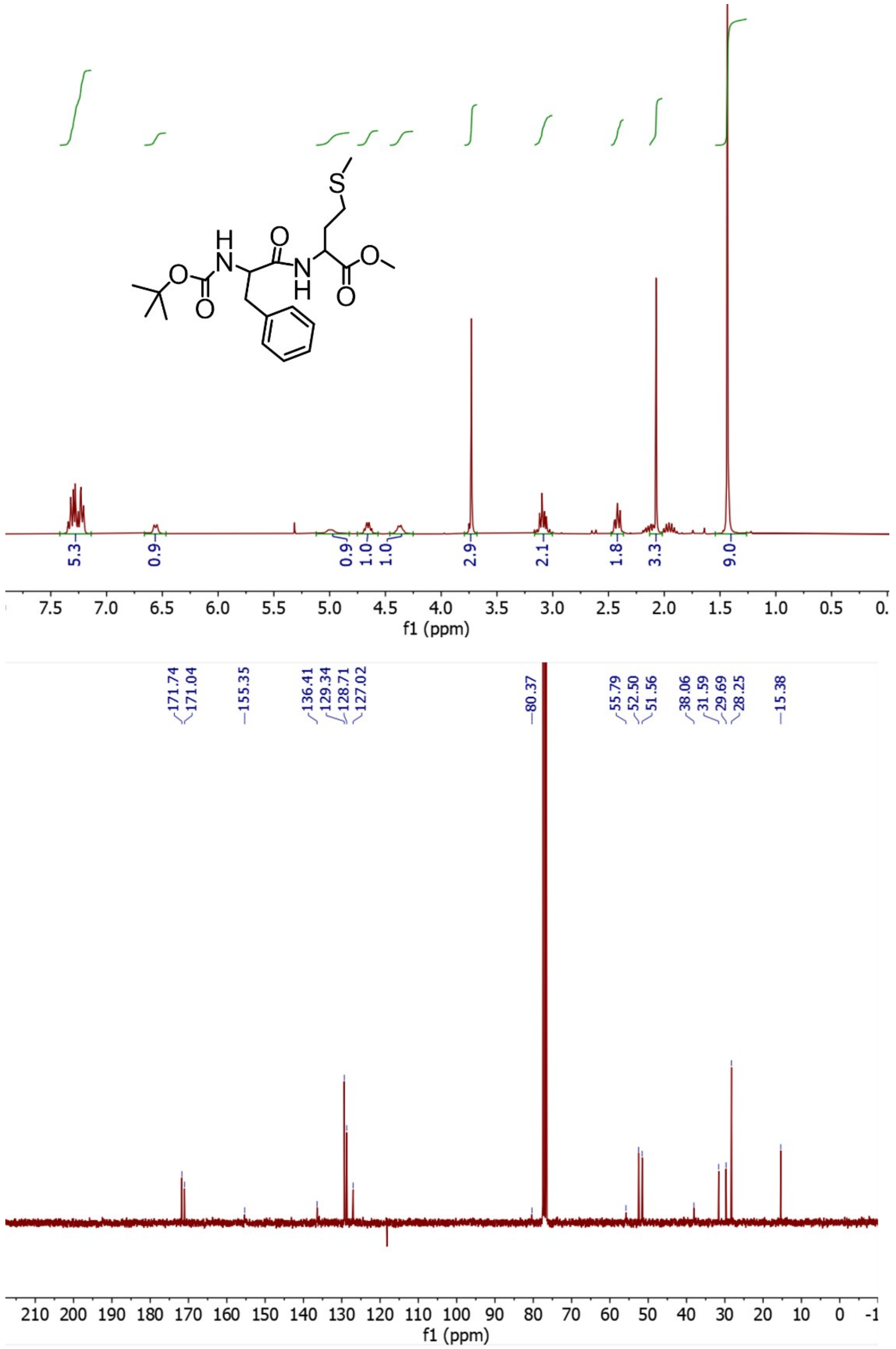

Figure S2. ^{1}H NMR and ^{13}C NMR spectra of BOC-F-S-OMe.

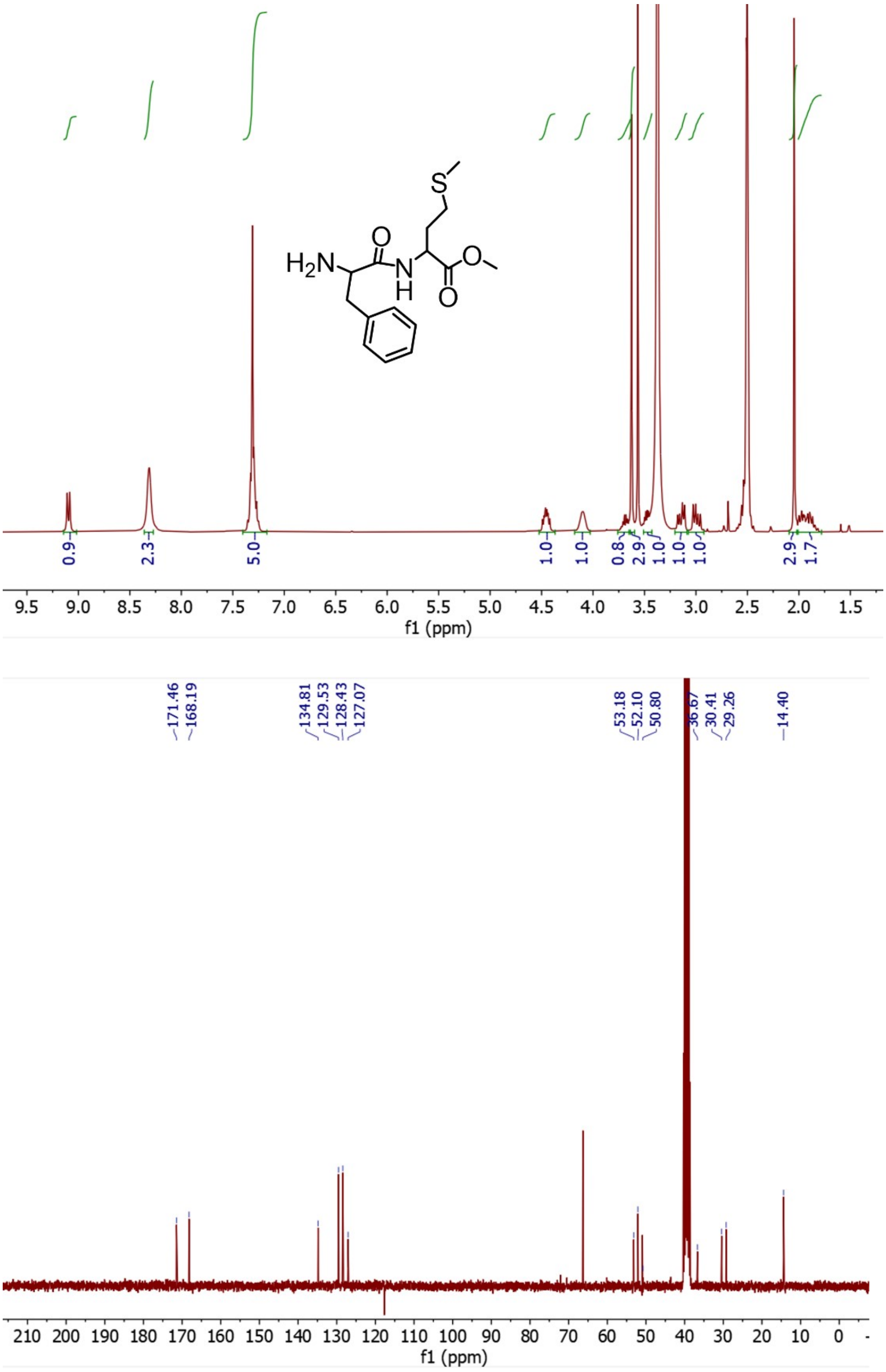

Figure S3. ^{1}H NMR and ^{13}C NMR spectra of NH_2-F-S-OMe.

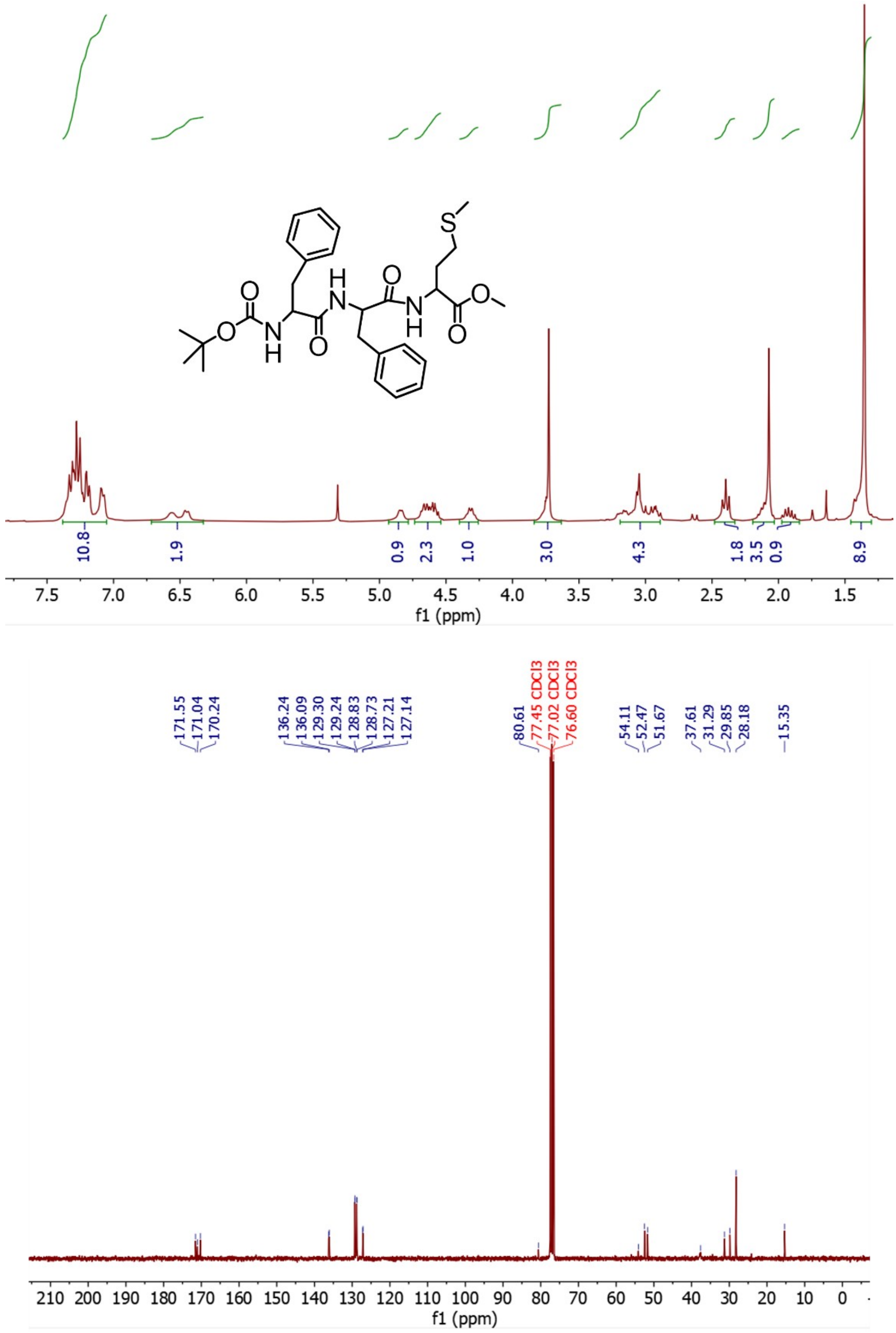

Figure S4. ^{1}H NMR and ^{13}C NMR spectra of BOC-F-F-S-OMe.

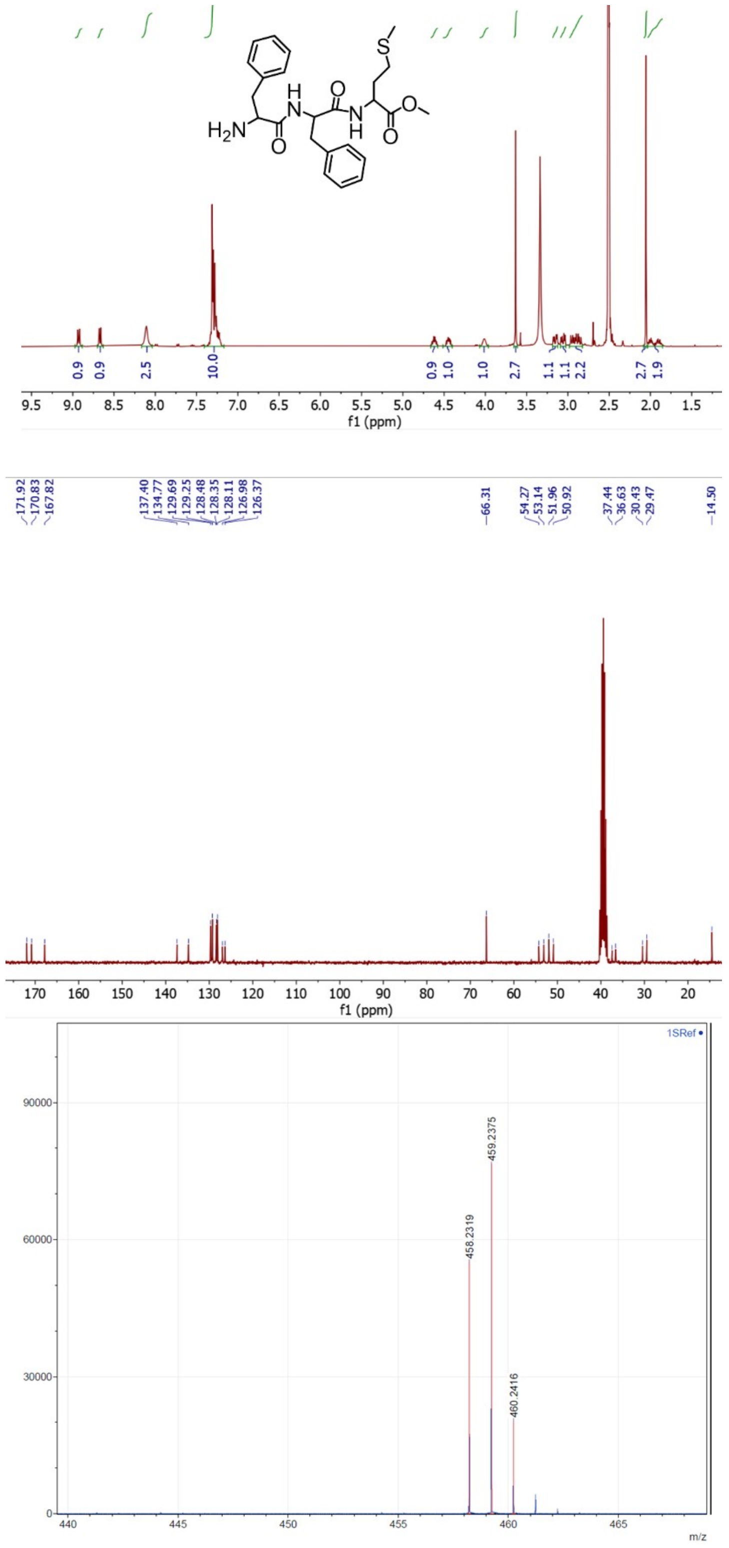

Figure S5. ^{1}H NMR, ^{13}C NMR spectra and MALDI-TOF spectrums of FFM.

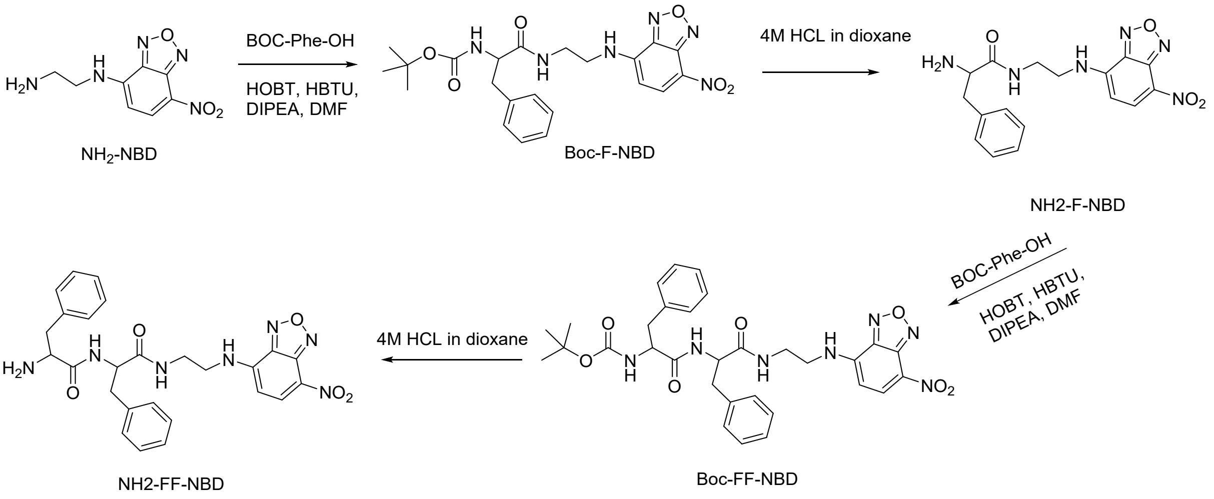

Figure S6. Synthesis procedure of NH2-FF-NBD.

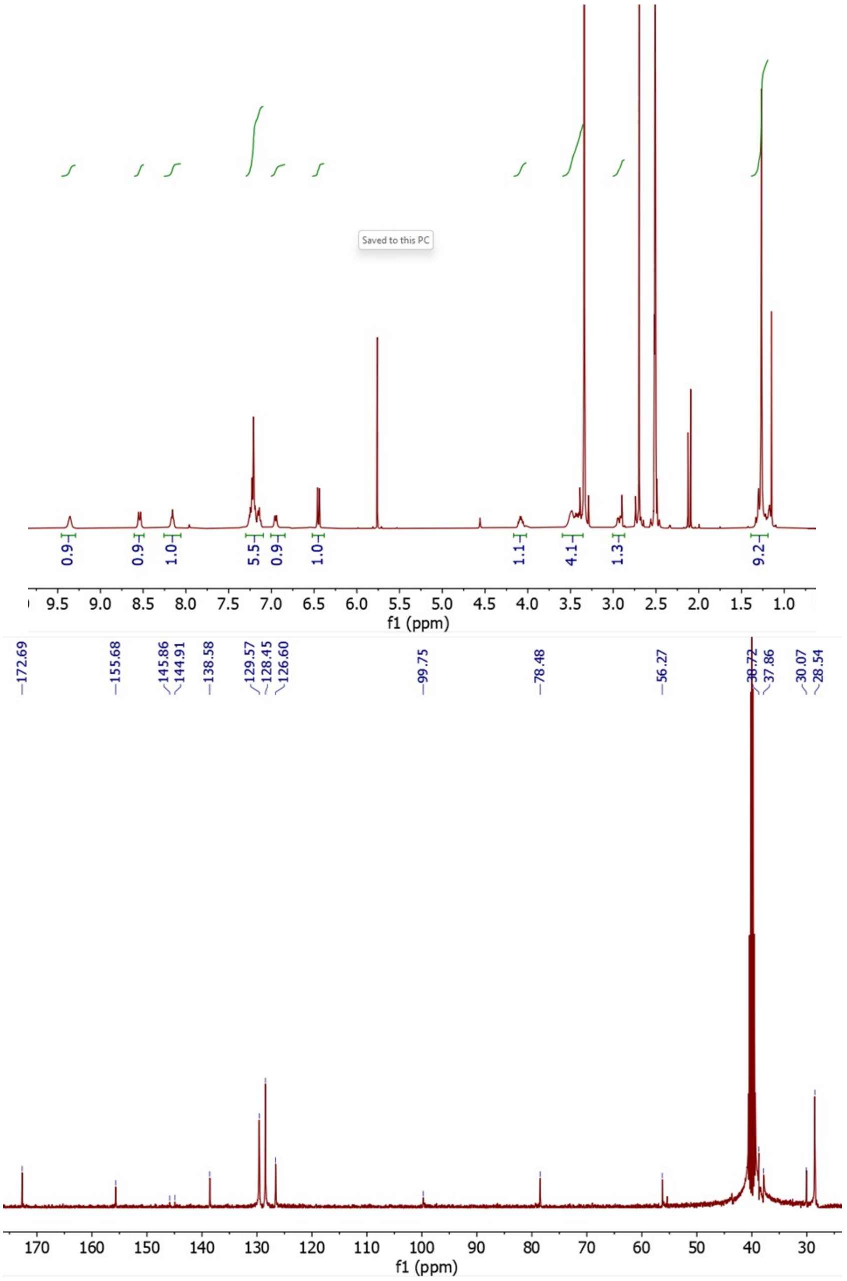

Figure S7. ^{1}H NMR and ^{13}C NMR spectra of Boc-F-NBD.

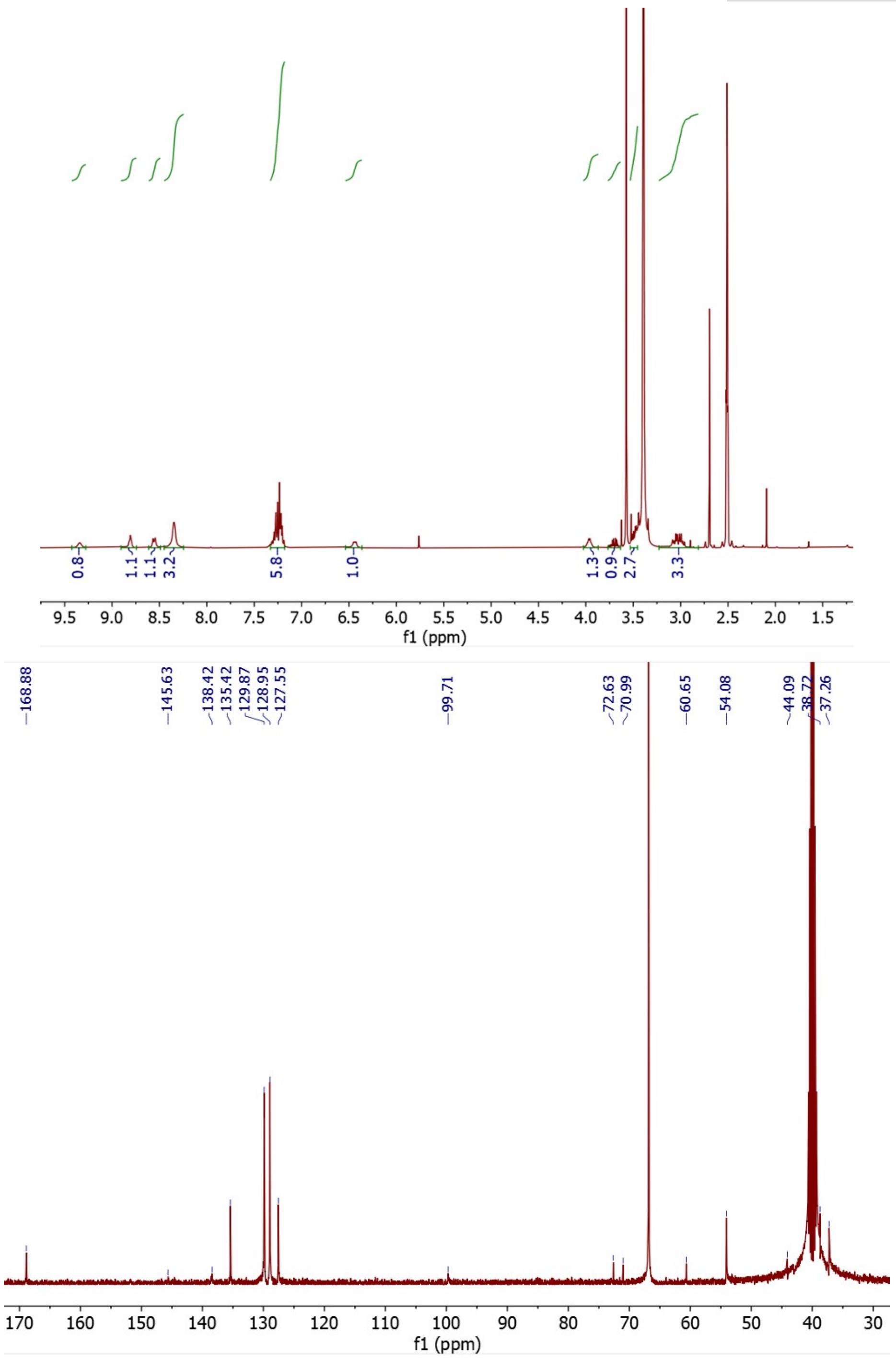

Figure S8. ^{1}H NMR and ^{13}C NMR spectra of NH_2-F-NBD.

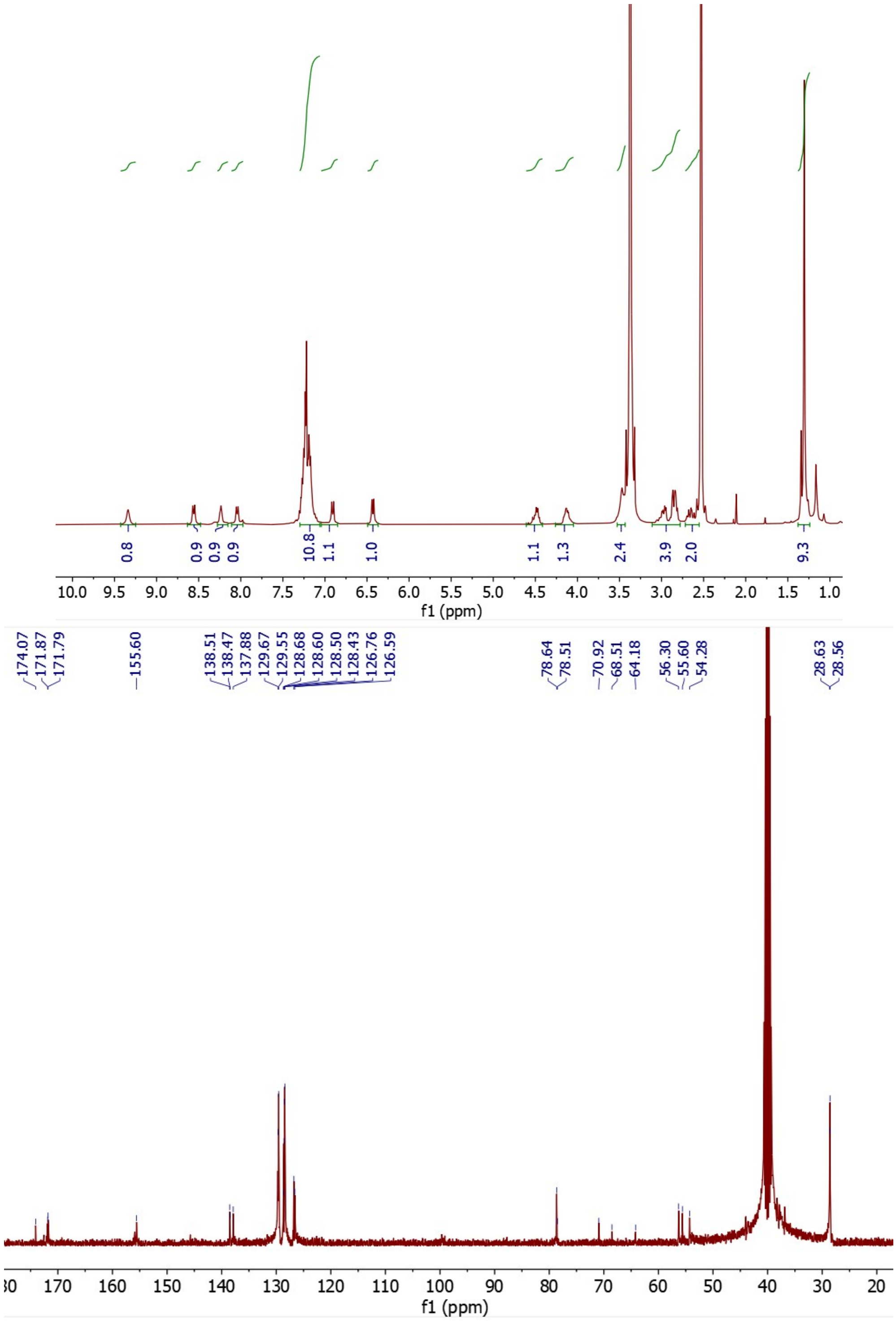

Figure S9. ^{1}H NMR and ^{13}C NMR spectra of BOC-FF-NBD.

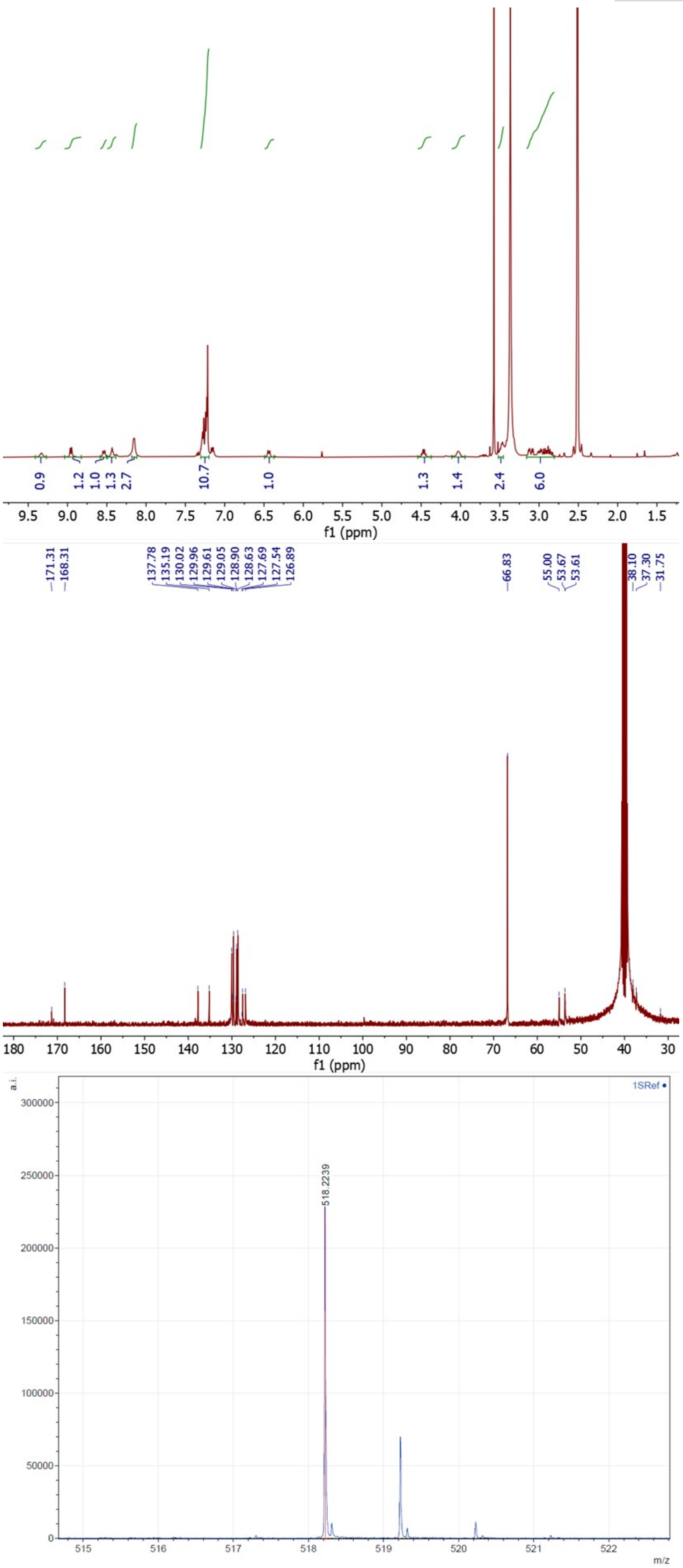

Figure S10. ^{1}H NMR, ^{13}C NMR and mass spectra of NH2-FF-NBD.

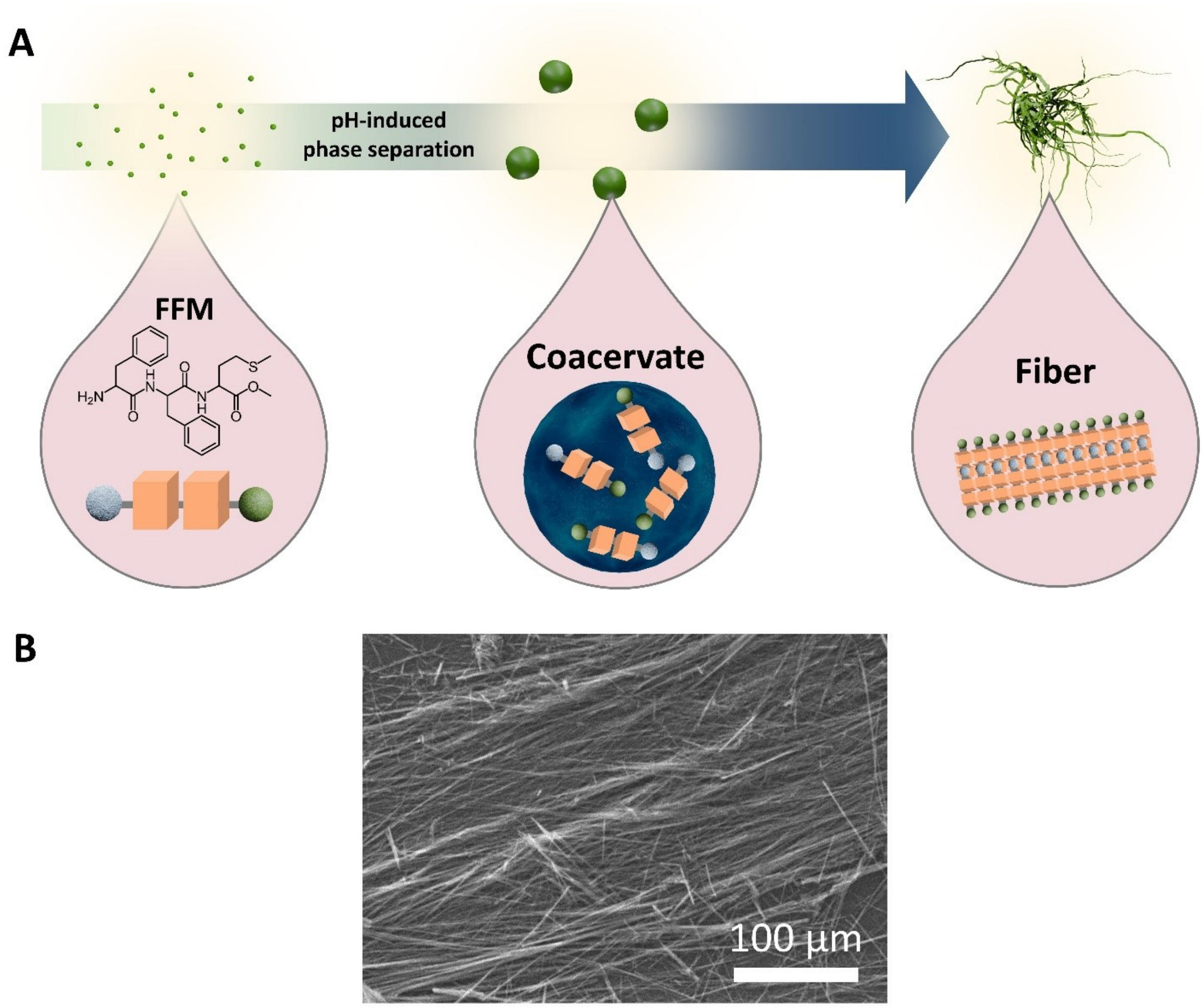

Figure S11. The assembly of peptide molecules into fibers in bulk. A. Schematic illustration of the fiber formation from liquid-liquid phase separation of FFM peptide. B. SEM micrographs of FFM fibers grown in bulk (FFM 6.67 mg/mL in HEPES 5 mM).

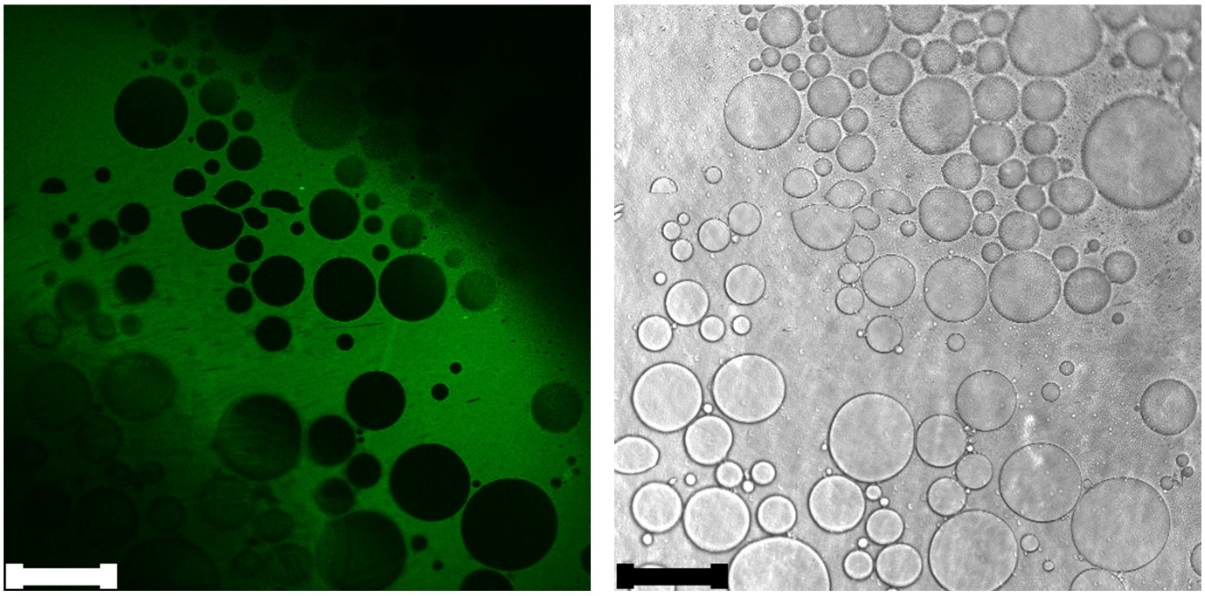

Figure S12. Water-in-oil emulsion with an aqueous phase composed of FFM and FF-NBD in HEPES buffer and an oil phase composed of PGPR in cyclohexane. FFM and FF-NBD diffused to the oil phase during emulsion formation.

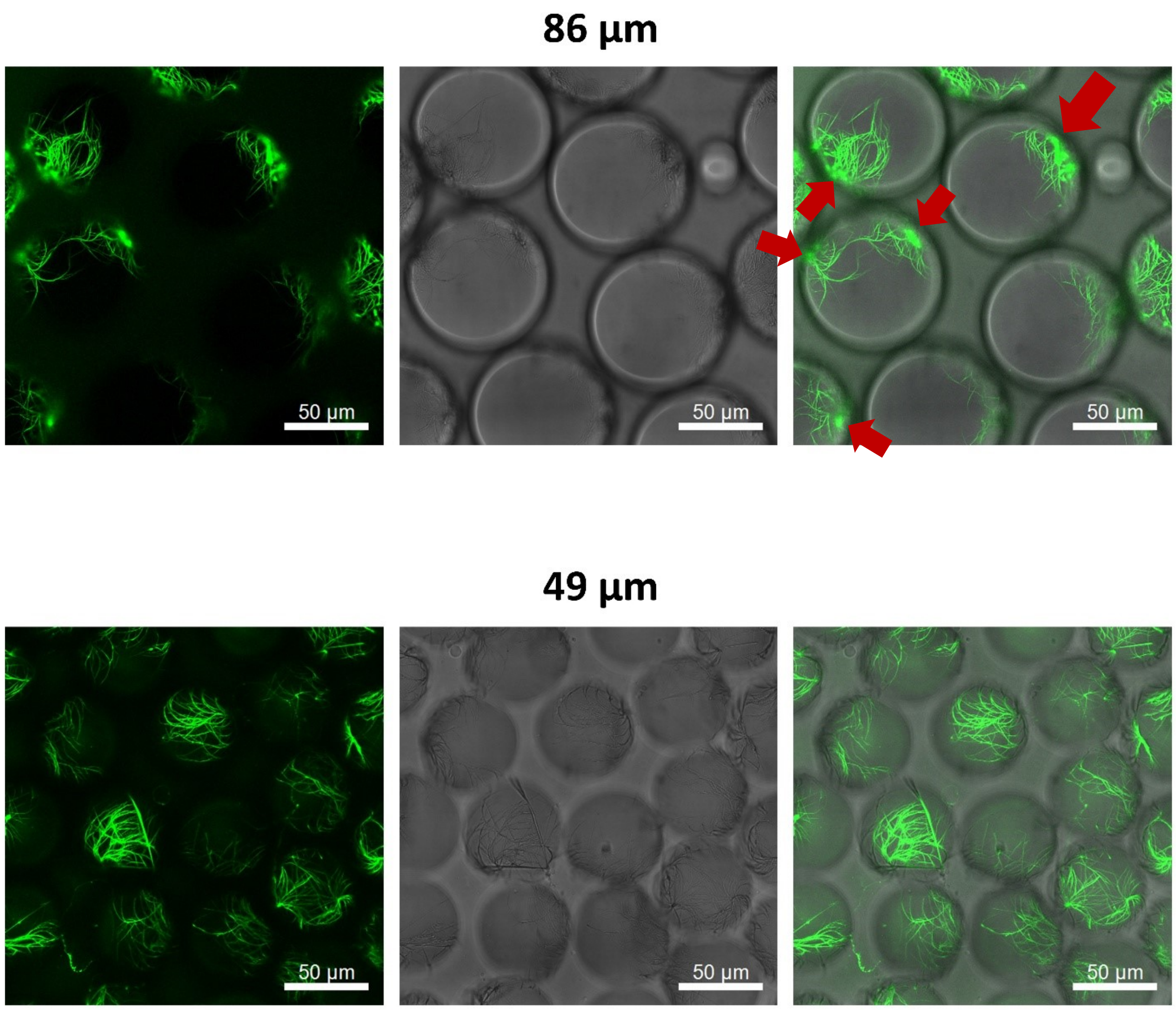

Figure S13. Fiber formation in droplets of different diameters. CLSM micrographs of FFM + FF-NBD fibers in droplets with diameters of 86 ± 4 µm and 49 ± 3 µm. Red arrows mark the nucleation sites where the fibers started to form and elongate.

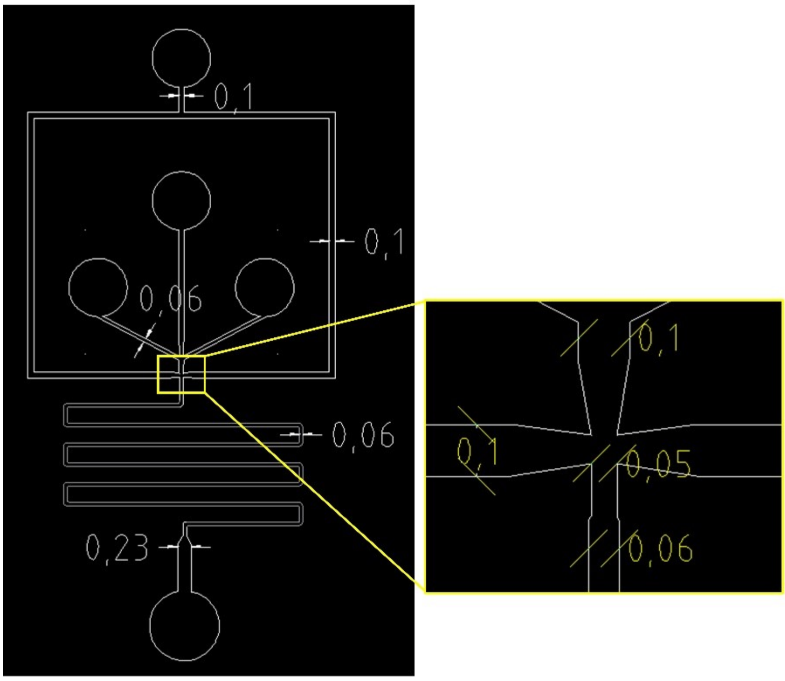

Figure S14. Droplet generation chip design. Unit: millimeter.

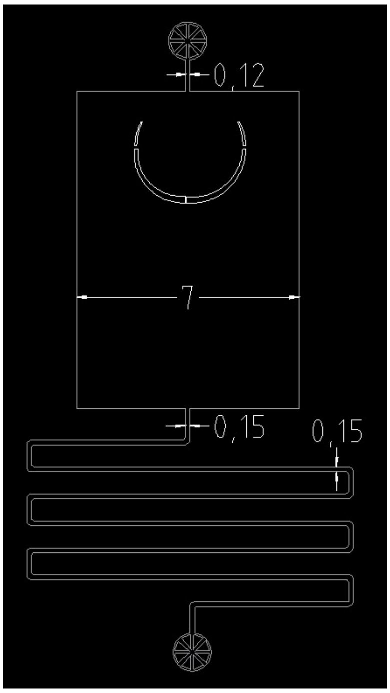

Figure S15. Trap chip design for observation under microscope. Unit: millimeter.

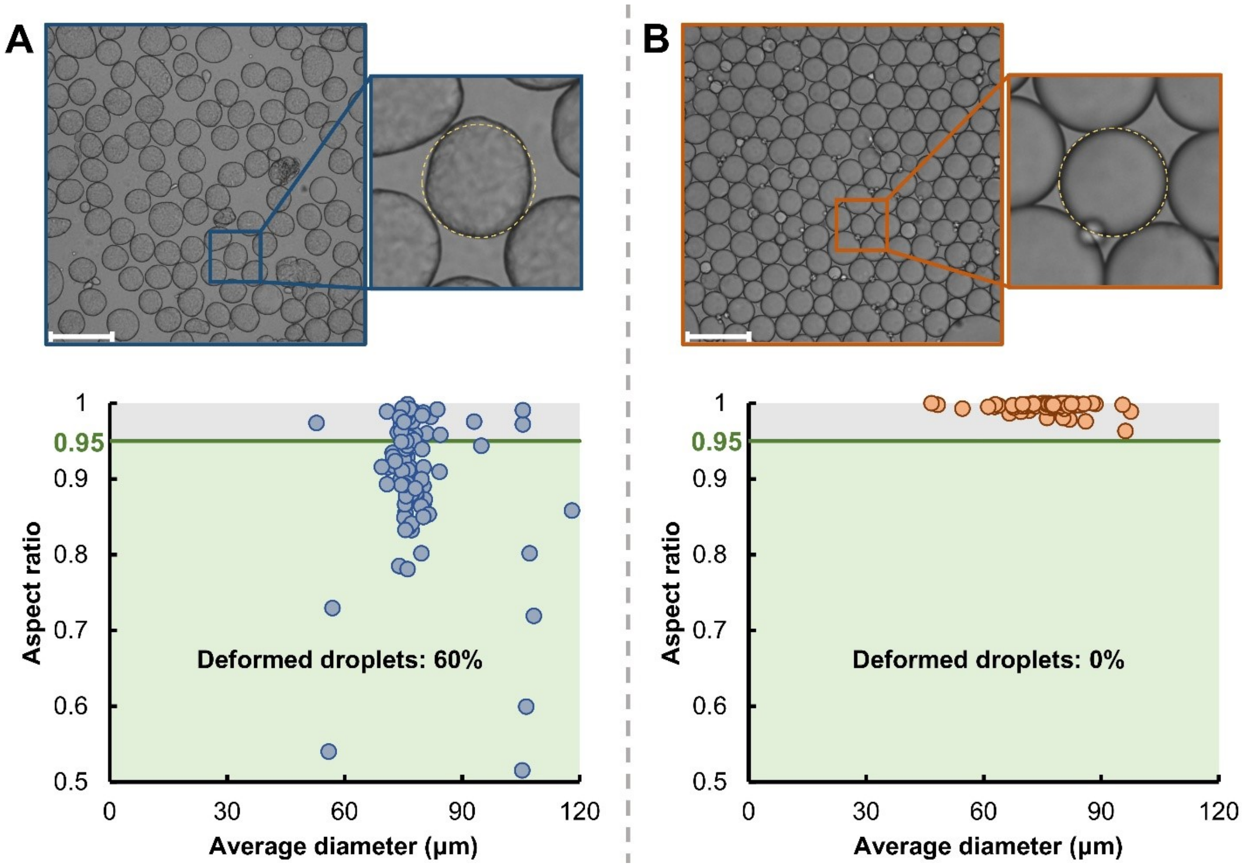

Figure S16. Deformation of droplets caused by the formation of the cytoskeleton. CLSM micrographs (top) and scatter plot (bottom) of the aspect ratio against average diameter of droplets prepared with (A) and without (B) FFM peptide fibers. Scale bar: 200 μm. The yellow-dashed circles represent the perimeters of spherical droplets with comparable area.

Table S1. Microfluidic setup to produce w/o emulsion containing FFM and FF-NBD fibers.

Solution	Component	Concentration	Flow rate (μL/h)
Inner fluid 1	FFM in HEPES 5mM (pH 5.3)	37.7 mg/mL	20
	FF-NBD in HEPES 5mM (pH 5.3)	1.1 mg/mL	
Inner fluid 2	HEPES (pH 12)	5mM	100
Outer fluid	FluoroSurfactant in HFE 7500	50 mg/mL	400

Table S2. Microfluidic setup to produce w/o emulsion containing FFM fibers and fluorescent-labeled enzymes.

Solution	Component	Concentration	Flow rate (μL/h)
Inner fluid 1	FFM in HEPES 5mM (pH 5.3)	37.6 mg/mL	20
	FITC-HRP	0.02 mg/mL	
	Cy5-GOx	0.07 mg/mL	
Inner fluid 2	HEPES (pH 12)	5mM	100
	NaCl	24 mg/mL	
Outer fluid	FluoroSurfactant in HFE 7500	50 mg/mL	400

Table S3. Composition of microreactors for the Gox-HRP cascade reaction.

Solution	Component	Concentration
Inner fluid 1 (aqueous phase)	FFM in HEPES 5mM (pH 5.3)	36.6 mg/mL
	FFM-NBD in HEPES 5mM (pH 5.3)	0.3 mg/mL
	HRP in PBS (pH 7)	0.02 mg/mL
	GOx in PBS (pH 7)	0.3 mg/mL
Inner fluid 2 (aqueous phase)	HEPES (pH 12.3)	5mM
	Glucose	0.1M
	Amplex red	0.08mM
	NaCl	24 mg/mL
Outer fluid (oil phase)	FluoroSurfactant in HFE 7500	50 mg/mL

Table S4. Microfluidic setup to produce different sizes of microreactors.

Droplet size (µm)	Flow rates (µL/h)		
	Inner fluid 1	Inner fluid 2	Outer fluid
86 ± 4	20	100	400
49 ± 3			1000
31 ± 2			2000

Theoretical surface area and surface area-to-volume ratio

The theoretical surface area and surface area-to-volume ratio of droplets with different sizes are calculated for every 100 μL aqueous phase as follows:

We assume that before the fiber formation, which causes droplet deformation, all droplets are spherical. d and r are the diameter and radius of the droplets, respectively.

Volume of one droplet: $V = \frac{4}{3}\pi r^3$

Number of droplets produced from 100 μL aqueous phase: $n = \frac{100}{V}$

Surface area of one droplet: $S = \pi d^2$

Total surface area: $S_{total} = S \times n$

Table S5. Diameter, radius and calculated volume of one droplet, number of droplets, surface area of one droplet, surface-area-to-volume ratio, and total surface area of droplets with different sizes.

Diameter (d), (μm)	Radius (r), (μm)	Volume of one droplet (V) ($μm^3$)	Number of droplets (n)	Surface area of one droplet (S), ($μm^2$)	Surface-area-to-volume (S/V) ($μm^{-1}$)	Total surface area (S_{total}) ($μm^2$)
86	43	333038	3.00×10^5	23235	0.07	6.98×10^9
49	24.5	61601	1.62×10^6	7543	0.12	1.22×10^{10}
31	15.5	15599	6.41×10^6	3019	0.19	1.94×10^{10}

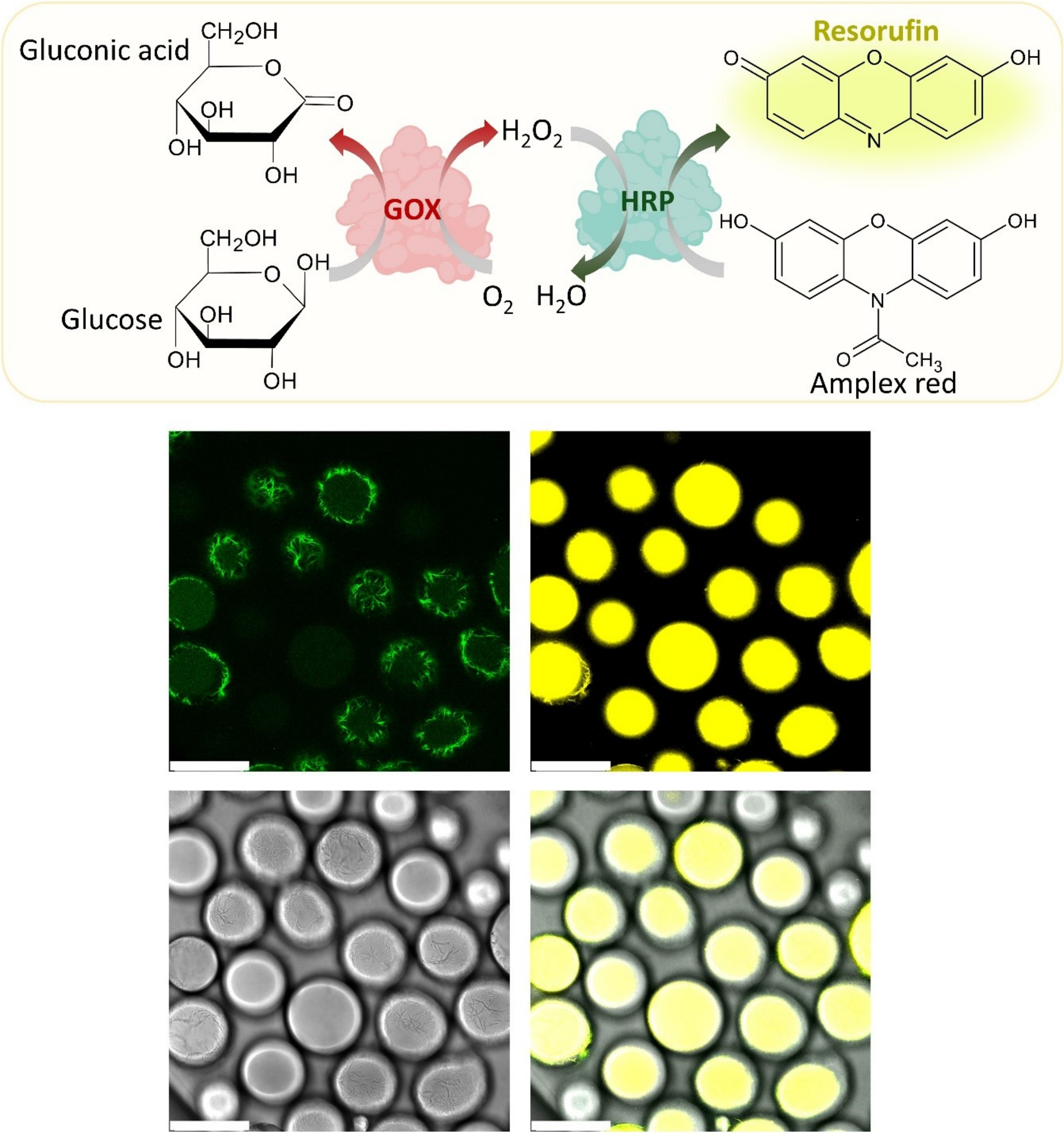

Figure S17. Cascade reaction in microreactor. Schematic illustration of GOx-HRP cascade reaction and CLSM micrographs of microreactors in which the reaction took place. Fluorescence signals of FF-NBD are shown in the green channel whereas fluorescence signals of resorufin are shown in the yellow channel.

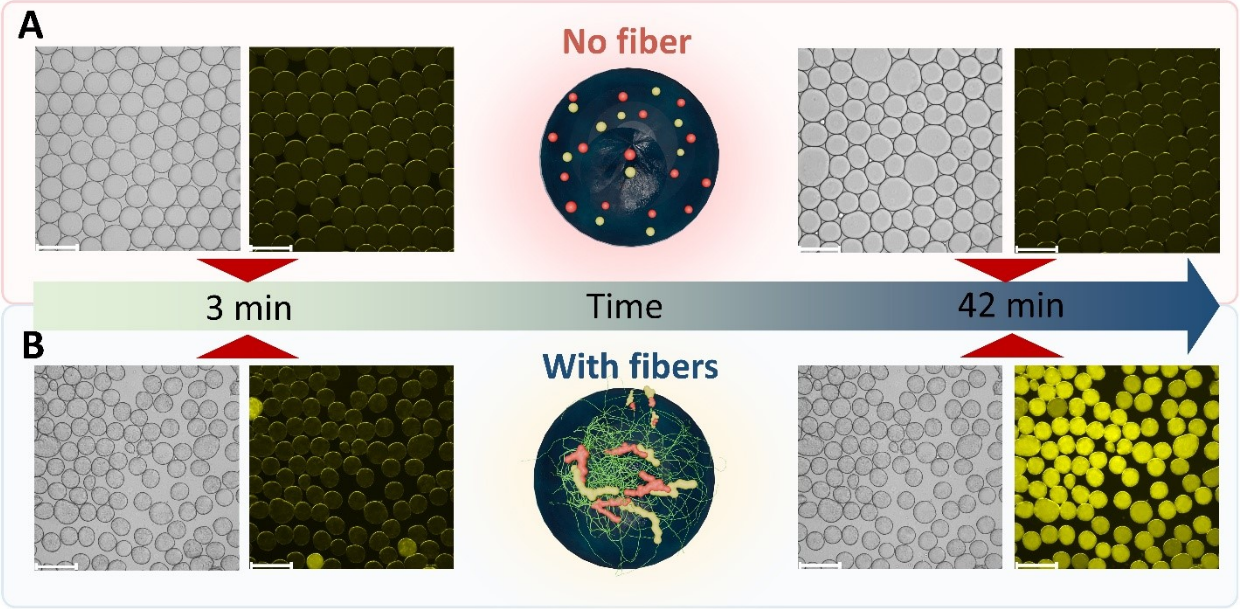

Figure S18. GOx-HRP cascade in microreactors with and without fibers. Micrographs from fluorescence microscope of the GOx-HRP cascade reaction in microreactors (A) without and (B) with the peptide cytoskeleton at 3 and 42 min. The glucose concentration was 83.3 µM. Scale bars: 200 µm.

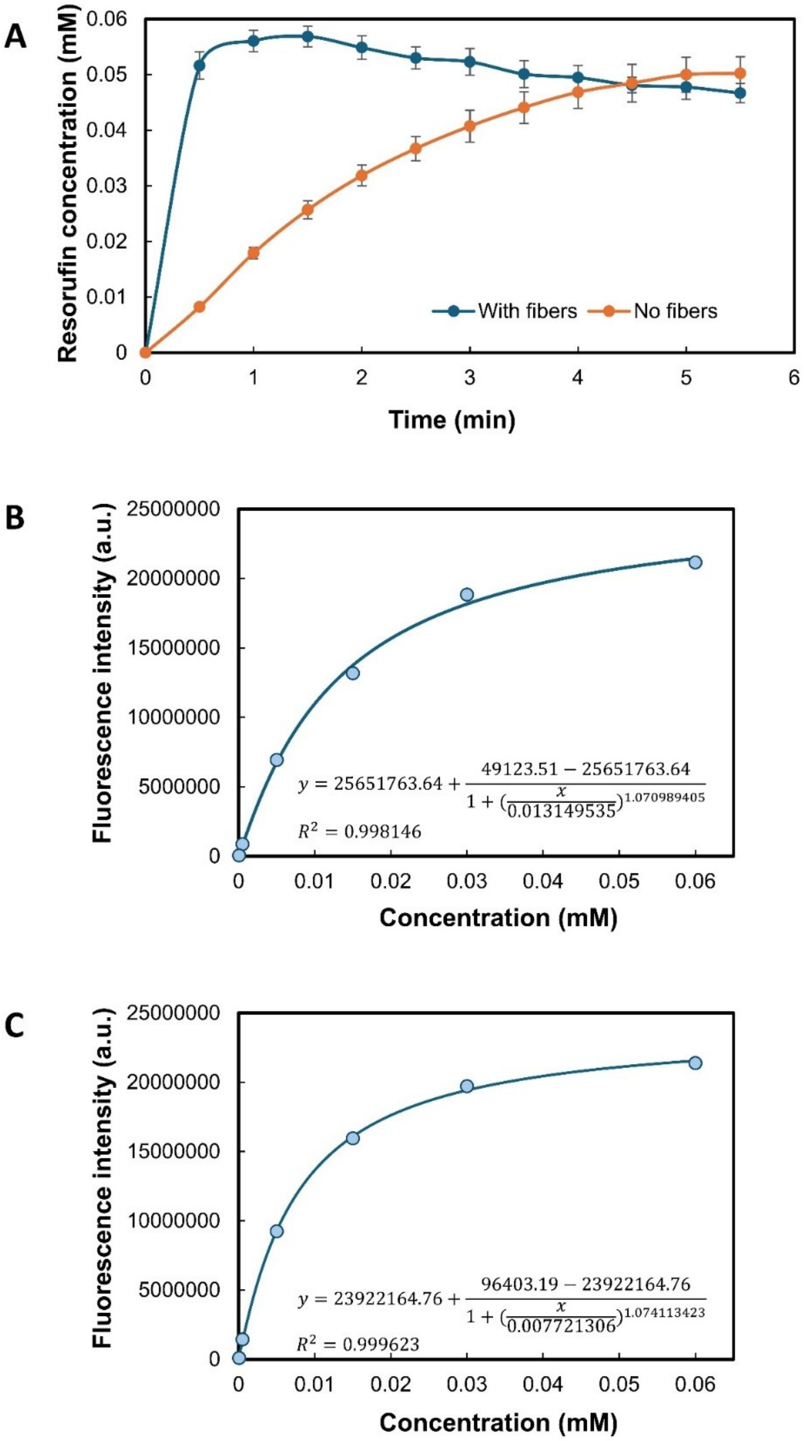

Figure S19. A. Kinetics of resorufin production in HEPES 5 mM solutions with or without fibers at pH 6.5. The glucose concentration was 200 mM. Error bars represent the standard deviation of 3 independently repeated experiments. B. Resorufin calibration curve at pH 6.5 in the presence of fibers. Error bars represent the standard deviation of 3 independently repeated experiments; those not visible are smaller than the symbol size. C. Resorufin calibration curve at pH 6.5 in the absence of fibers. Error bars represent the standard deviation of 4 independently repeated experiments; those not visible are smaller than the symbol size.

Supporting movies

Movie S1. 3D reconstruction of microcompartments with artificial cytoskeleton prepared with FFM and FluoroSurfactant 5 mg/mL in HFE 7500 oil.

Movie S2. Stability of microcompartments with artificial cytoskeleton prepared with FFM and FluoroSurfactant 5 mg/mL in HFE 7500 oil over 5.5 hours. The video is displayed at 1260-fold acceleration. The movement of all droplets toward the left side of the field at later time points is due to evaporation over the long observation period.

Movie S3. Cascade reaction GOx-HRP with a glucose concentration of 200 mM in microreactors with (right) or without (left) cytoskeleton. Time-lapse movie showing the reaction progression, with frames acquired every 3 min from the 3rd to the 33rd minute.

Movie S4. Cascade reaction GOx-HRP with a glucose concentration of 83.3 mM in microreactors with (right) or without (left) cytoskeleton. Time-lapse movie showing the reaction progression, with frames acquired every 3 min from the 5th to the 44th minute.

Movie S5. Cascade reaction GOx-HRP with a glucose concentration of 5 mM in microreactors with a diameter of 86 (left), 49 (middle) and 31 μm (right). Time-lapse movie showing the reaction progression, with frames acquired every 3 min from the 9th to the 100th minute.

References

1. S. Cao, T. Ivanov, J. Heuer, C. T. J. Ferguson, K. Landfester and L. Caire da Silva, *Nature Communications*, 2024, **15**, 39.
2. A. Bandyopadhyay, S. Cambray and J. Gao, *Journal of the American Chemical Society*, 2017, **139**, 871-878.
3. M. Abbas, W. P. Lipiński, K. K. Nakashima, W. T. S. Huck and E. Spruijt, *Nature Chemistry*, 2021, **13**, 1046-1054.
